# Pre-B Cell Receptor Signaling Induces Immunoglobulin κ Locus Accessibility by Functional Redistribution of Enhancer-Mediated Chromatin Interactions

**DOI:** 10.1371/journal.pbio.1001791

**Published:** 2014-02-18

**Authors:** Ralph Stadhouders, Marjolein J. W. de Bruijn, Magdalena B. Rother, Saravanan Yuvaraj, Claudia Ribeiro de Almeida, Petros Kolovos, Menno C. Van Zelm, Wilfred van Ijcken, Frank Grosveld, Eric Soler, Rudi W. Hendriks

**Affiliations:** 1 Department of Cell Biology, Erasmus MC Rotterdam, The Netherlands; 2 Department of Pulmonary Medicine, Erasmus MC Rotterdam, The Netherlands; 3 Department of Immunology, Erasmus MC Rotterdam, The Netherlands; 4 Center for Biomics, Erasmus MC Rotterdam, The Netherlands; 5 The Cancer Genomics Center, Erasmus MC Rotterdam, The Netherlands; 6 INSERM UMR967 and French Alternative Energies and Atomic Energy Commission (CEA), Fontenay-aux-Roses, France; Scripps Research Institute, United States of America

## Abstract

During B cell development, the precursor B cell receptor (pre-BCR) checkpoint is thought to increase immunoglobulin *κ* light chain (*Igκ*) locus accessibility to the V(D)J recombinase. Accordingly, pre-B cells lacking the pre-BCR signaling molecules Btk or Slp65 showed reduced germline V_κ_ transcription. To investigate whether pre-BCR signaling modulates V_κ_ accessibility through enhancer-mediated *Igκ* locus topology, we performed chromosome conformation capture and sequencing analyses. These revealed that already in pro-B cells the κ enhancers robustly interact with the ∼3.2 Mb V_κ_ region and its flanking sequences. Analyses in wild-type, Btk, and Slp65 single- and double-deficient pre-B cells demonstrated that pre-BCR signaling reduces interactions of both enhancers with *Igκ* locus flanking sequences and increases interactions of the 3′κ enhancer with V_κ_ genes. Remarkably, pre-BCR signaling does not significantly affect interactions between the intronic enhancer and V_κ_ genes, which are already robust in pro-B cells. Both enhancers interact most frequently with highly used V_κ_ genes, which are often marked by transcription factor E2a. We conclude that the κ enhancers interact with the V_κ_ region already in pro-B cells and that pre-BCR signaling induces accessibility through a functional redistribution of long-range chromatin interactions within the V_κ_ region, whereby the two enhancers play distinct roles.

## Abstract 2

Chromatin conformation analyses provide novel insights into how variable segments in the immunoglobulin light chain gene become accessible for recombination in precursor B lymphocytes.

## Author Summary

B lymphocyte development involves the generation of a functional antigen receptor, comprising two heavy chains and two light chains arranged in a characteristic “Y” shape. To do this, the receptor genes must first be assembled by ordered genomic recombination events, starting with the immunoglobulin heavy chain (IgH) gene segments. On successful rearrangement, the resulting IgH μ protein is presented on the cell surface as part of a preliminary version of the B cell receptor—the “pre-BCR.” Pre-BCR signaling then redirects recombination activity to the immunoglobulin κ light chain gene. The activity of two regulatory κ enhancer elements is known to be crucial for opening up the gene, but it remains largely unknown how the hundred or so Variable (V) segments in the κ locus gain access to the recombination system. Here, we studied a panel of pre-B cells from mice lacking specific signaling molecules, reflecting absent, partial, or complete pre-BCR signaling. We identify gene regulatory changes that are dependent on pre-BCR signaling and occur via long-range chromatin interactions between the κ enhancers and the V segments. Surprisingly the light chain gene initially contracts, but the interactions then become more functionally redistributed when pre-BCR signaling occurs. Interestingly, we find that the two enhancers play distinct roles in the process of coordinating chromatin interactions towards the V segments. Our study combines chromatin conformation techniques with data on transcription factor binding to gain unique insights into the functional role of chromatin dynamics.

## Introduction

B lymphocyte development is characterized by stepwise recombination of immunoglobulin (Ig), variable (V), diversity (D), and joining (J) genes, whereby in pro-B cells the Ig heavy (H) chain locus rearranges before the *Igκ* or *Igλ* light (L) chain loci [1],[2]. Productive *IgH* chain rearrangement is monitored by deposition of the *IgH* μ chain protein on the cell surface, together with the preexisting surrogate light chain (SLC) proteins λ5 and VpreB, as the pre-B cell receptor (pre-BCR) complex [3]. Pre-BCR expression serves as a checkpoint that monitors for functional *IgH* chain rearrangement, triggers proliferative expansion, and induces developmental progression of large cycling into small resting Ig μ^+^ pre-B cells in which the recombination machinery is reactivated for rearrangement of the *Igκ* or *Igλ* L chain loci [3],[4].

During the V(D)J recombination process, the spatial organization of large antigen receptor loci is actively remodeled [5]. Overall locus contraction is achieved through long-range chromatin interactions between proximal and distal regions within these loci. This process brings distal V genes in close proximity to (D)J regions, to which Rag (recombination activating gene) protein binding occurs [6] and the nearby regulatory elements that are required for topological organization and recombination [5],[7],[8]. The recombination-associated changes in locus topology thereby provide equal opportunities for individual V genes to be recombined to a (D)J segment. Accessibility and recombination of antigen receptor loci are controlled by many DNA-binding factors that interact with local *cis*-regulatory elements, such as promoters, enhancers, or silencers [7]–[9]. The long-range chromatin interactions involved in this process are thought to be crucial for the regulation of V(D)J recombination and orchestrate changes in subnuclear relocation, germline transcription, histone acetylation and/or methylation, DNA demethylation, and compaction of antigen receptor loci [5],[10].

The mouse *Igκ* locus harbors 101 functional V_κ_ genes and four functional J_κ_ elements and is spread over >3 Mb of genomic DNA [11]. Mechanisms regulating the site-specific DNA recombination reactions that create a diverse *Igκ* repertoire are complex and involve local differences in the accessibility of the V_κ_ and J_κ_ genes to the recombinase proteins [12]. Developmental-stage-specific changes in gene accessibility are reflected by germline transcription, which precedes or accompanies gene recombination [13]. In the *Igκ* locus, germline transcription is initiated from promoters located upstream of J_κ_ (referred to as κ^0^ transcripts) and from V_κ_ promoters [14]. Deletion of the intronic enhancer (iEκ), located between J_κ_ and C_κ_, or the downstream 3′κ enhancer (3′Eκ), both containing binding sites for the E2a and Irf4/Irf8 transcription factors (TFs), diminishes *Igκ* locus germline transcription and recombination [15]–[19]. On the other hand, the Sis (silencer in intervening sequence) element in the V_κ_–J_κ_ region negatively regulates *Igκ* rearrangement [20]. This Sis element was shown to target *Igκ* alleles to centromeric heterochromatin and to associate with the Ikaros repressor protein that also colocalizes with centromeric heterochromatin. Sis contains a strong binding site for the zinc-finger transcription regulator CTCC-binding factor (Ctcf) [21],[22]. Interestingly, deletion of the Sis element or conditional deletion of the *Ctcf* gene in the B cell lineage both resulted in reduced κ^0^ germline transcription and enhanced proximal V_κ_ usage [21],[23]. Very recently, a novel Ctcf binding element located directly upstream of the Sis region was shown to be essential for locus contraction and recombination to distal V_κ_ genes [23]. In addition, the *Igκ* repertoire is controlled by the polycomb group protein YY1 [24].

Induction of *Igκ* rearrangements requires the expression of the Rag1 and Rag2 proteins, the attenuation of the cell cycle, and transcriptional activation of the *Igκ* locus, all of which are thought to be crucially dependent on pre-BCR signaling [4],[25]. At first, pre-BCR signals synergize with interleukin-7 receptor (IL-7R) signals to drive proliferative expansion of IgH μ^+^ large pre-B cells [4]. In these cells, transcription of the *Rag* genes is low and the Rag2 protein is unstable due to cell-cycle-dependent degradation [26]. Subsequently, signaling through the pre-BCR downstream adapter Slp65 (SH2-domain-containing leukocyte protein of 65 kDa, also known as Blnk or Bash) switches cell fate from proliferation to differentiation [4]. Importantly, Slp65 (i) induces the TF Aiolos, which down-regulates λ5 expression [27]; (ii) binds Jak3 and thereby interferes with IL-7R signaling [28]; and (iii) reduces inhibitory phosphorylation of Foxo TFs [29]. All these changes result in attenuation of the cell cycle and thus Rag protein stabilization. Moreover, *Rag* gene transcription is induced by Foxo proteins [30].

Although rearrangement and expression of the *Igκ* locus can occur independently of IgH μ chain expression [31],[32], several lines of evidence indicate that pre-BCR signaling is actively involved in inducing *Igκ* and *Igλ* locus accessibility and gene rearrangement. First, surface IgH μ chain expression correlates with germline transcription in the *Igκ* locus [33]. Second, in the absence of Slp65, κ^0^ germline transcription is reduced [34]. Third, mice deficient for Bruton's tyrosine kinase (Btk), which is a pre-BCR downstream signaling molecule interacting with Slp65, show reduced *Igλ* L chain germline transcription and reduced Igλ usage [35]. Fourth, transgenic expression of the constitutively active E41K-Btk mutant in IgH μ chain negative pro-B cells induces premature rearrangement and protein expression of Igκ L chain [34]. Based on fluorescence in situ hybridization (FISH) studies, it has been proposed that in pro-B cells distal Vκ and Cκ genes are separated by large distances and that the *Igκ* locus specifically undergoes contraction in small pre-B and immature B cells actively undergoing V_κ_-J_κ_ recombination [36]. However, it remains unknown how pre-BCR-induced signals affect the accessibility, contraction, and topology of the Vκ region, or how they affect the long-range interactions of the κ regulatory elements involved in organizing these events.

In this study, we identified the effects of pre-BCR signaling on germline Vκ transcription and on the expression of TFs implicated in the regulation of *Igκ* gene rearrangement. We found that the decrease in pre-BCR signaling capacity in wild-type, Btk-deficient, Slp65-deficient, and Btk/Slp65 double-deficient pre-B cells was paralleled by a gradient of decreased expression of many TFs including Ikaros, Aiolos, Irf4, and (to a lesser extent) E2a, as well as by a decreased *Igκ* locus accessibility for recombination. Several of these factors can mediate long-range chromatin interactions and are known to occupy κ regulatory elements that regulate locus accessibility [37]–[40]. We therefore sought to analyze the effect of pre-BCR signaling on the higher order chromatin structure organized by these regulatory sequences at the *Igκ* locus. To this end, we performed chromosome conformation capture and sequencing (3C-seq) analyses [41] on pro-B cells and pre-B cells from mice single or double deficient for Btk or Slp65 to evaluate the effects of this pre-BCR signaling gradient on *Igκ* locus topology. These 3C-seq experiments demonstrated that already in pro-B cells the κ enhancers robustly interact with the ∼3.2 Mb Vκ region and its flanking sequences, and that pre-BCR signaling induces accessibility by a functional redistribution of enhancer-mediated chromatin interactions within the V_κ_ region.

## Results

### Identification of Genes Regulated by Pre-BCR Signaling

**Figure 1:**
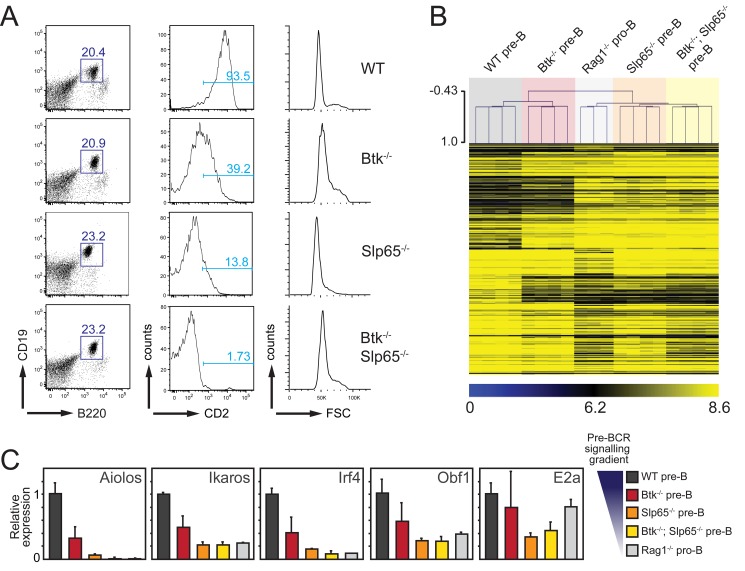
Gene expression profiling strategy for the identification of genes regulated by Btk/Slp65-mediated pre-BCR signaling. (A) FACS sorting strategy for purification of pre-B cell fractions from the indicated mice on a VH81x transgenic *Rag1* ^−/−^ background. Lymphocytes were gated on the basis of forward/side scatter and B220^+^CD19^+^ pre-B cell fractions were sorted. Virtually all B220^+^CD19^+^ cells were cytoplasmic μ heavy chain positive [34], but showed genotype-dependent levels of expression of the CD2 differentiation marker, in agreement with previous findings [34]. (B) DNA microarray analysis of total mRNA from FACS-purified B220^+^CD19^+^ pre-B/pro-B cell fractions from the indicated mice. One-way ANOVA analysis (*p* = 0.01) identified 266 significantly different genes. MeV hierarchical clustering of gene expression differences are represented in the heatmap. (C) Validation of the expression of TFs implicated in *Igκ* gene rearrangement. Total mRNA isolated from FACS-sorted B220^+^CD19^+^ pre-B/pro-B cell fractions from the indicated mice was analyzed by quantitative RT-PCR for expression of TFs. Expression levels were normalized to those of *Gapdh*, whereby the values in WT pre-B cells were set to one. Bars represent mean values and error bars denote standard deviations for four independent mice per group.

Whereas mice deficient for the pre-BCR signaling molecules Btk and Slp65 have a partial block at the pre-B cell stage [42],[43], in Btk/Slp65 double-deficient mice, only very few pre-B cells show progression to the immature B cell stage characterized by functional *IgL* chain gene recombination [44]. To enable analysis of the effects of pre-BCR signaling on (i) the expression of genes involved in *Igκ* gene rearrangement and on (ii) long-distance chromatin interactions in the *Igκ* locus in pre-B cells in the absence of *Igκ* gene recombination events, we bred Btk and Slp65 single- and double-deficient mice on the *Rag1* ^−/−^ background. In these mice, progression of B cell progenitors to the pre-B cell stage was conferred by the transgenic, functionally rearranged VH81x IgH μ chain, which ensures pre-BCR expression and cellular proliferation. The absence of functional Rag1 protein precludes *IgL* chain gene rearrangement and cells are completely arrested at the small pre-B cell stage (Figure 1A).

We performed genome-wide expression profiling of FACS-purified B220^+^CD19^+^ pre-B cell fractions from wild-type (WT), Btk, and Slp65 single- and double-deficient VH81x transgenic *Rag1* ^−/−^ mice (Figure 1A). In these experiments non-VH81x transgenic *Rag1* ^−/−^ pro-B cells served as controls. One-way ANOVA analysis using MeV software (*p*<0.01) [45] revealed that 266 genes were differentially expressed between the five groups of pro-B/pre-B cells (Figure 1B). When compared with WT VH81x transgenic *Rag1* ^−/−^ pre-B cells, 174 genes were up-regulated, whereby the average values of the fold increase were ∼1.70, ∼3.28, ∼3.36, and ∼3.47 for *Btk* ^−/−^, *Slp65* ^−/−^, *Btk* ^−/−^ *Slp65* ^−/−^ VH81x transgenic *Rag1* ^−/−^ pre-B cells and non-VH81x transgenic *Rag1* ^−/−^ pro-B cells, respectively (see Table S1). A similar gradient of gene expression changes was apparent from the average values of the fold change for the 192 significantly down-regulated genes, which were ∼1.65, ∼2.29, ∼3.79, and ∼4.15 in the four groups of pre-B/pro-B cells, respectively (see Table S2). In a hierarchical clustering analysis of the five groups of B cell precursors, the expression profiles of *Btk* ^−/−^ *Slp65* ^−/−^ VH81x transgenic *Rag1* ^−/−^ pre-B cells and non-VH81x transgenic *Rag1* ^−/−^ pro-B cells were very similar (Figure 1B). This implies that expression of the 266 genes is not substantially influenced by pre-BCR-mediated proliferation, which is still induced in pre-B cells lacking both Btk and Slp65 [44],[46] but not in *Rag1* ^−/−^ pro-B cells. Consistent with these findings, gene distance matrix analysis revealed a clear gene expression gradient among the five groups of pre-B/pro-B cells, in which *Btk* ^−/−^ *Slp65* ^−/−^ pre-B and *Rag1* ^−/−^ pro-B cells again showed highly comparably expression signatures (Figure S1).

In agreement with previous findings [34],[43],[46], pre-BCR signaling-defective pre-B cells manifested increased expression of *Dntt*, encoding terminal deoxynucleotidyl transferase and the SLC components Vpre (*Vpreb1*) and λ5 (*Igll1*), as well as decreased expression of the cell surface markers Cd2, Cd22, Cd25(IL-2R), and MHC class II (Table 1). *Btk* and *Slp65* single-deficient and particularly double-deficient pre-B cells failed to up-regulate various genes known to be involved in *IgL* chain recombination, such as *Ikzf3* (Aiolos), *Ikzf1* (Ikaros), *Irf4*, *Spib*, *Pou2f2* (Oct2), polymerase-μ [47], as well as *Hivep1* encoding the Mbp-1 protein, which has been shown to bind to the κ enhancers [48]. In addition, pre-BCR signaling influenced the expression levels of many other DNA-binding or modifying factors that were not previously associated with *IgL* chain recombination, including *Lmo4*, *Zfp710*, *Arid1a/3a/3b*, the lysine-specific demethylases *Aof1* and *Phf2*, *Prdm2* (a H3K9 methyltransferase), the *sik1* gene encoding a histon deacetylase (HDAC) kinase, *Hdac5*, *Hdac8*, and the DNA repair protein gene *Rev1* (Table S2). We did not find significant differences in the expression of several other TFs implicated in Ig gene recombination—for example, Obf1/Oca-B, Pax5, E2a, and Irf8 (Table 1). In addition, in signaling-deficient pre-B cells, we found reduced transcription of genes encoding several signaling molecules (e.g., *Rasgrp1*, *Rapgefl1*, *Ralgps2*, *Blk*, *Traf5*, *Hck*, *Nfkbia* (IκBα), *Syk*, *Csk*), cell surface markers (Cd38, Cd72, Cd74, Cd55, and Notch2), or genes regulating cell survival (*Bmf* and *Bcl2l1* encoding Bcl_XL_) (Table S2). Interestingly, we observed concomitant up-regulation of signaling molecules that are also associated with the T cell receptor (*Lat*, *Zap70*, and *Prkcq* (PKCθ); Table S1).

**Table 1:** Genes differentially expressed between WT, Btk, or Slp65 single or double mutant V_H_81X Tg *Rag1* ^−/−^ pre-B cells or *Rag1* ^−/−^ pro-B cells.

ID Probe Set	Accession Number	Gene	Description of Function	*p* Value^a^	Fold Change (Btk KO^b^)	Fold Change (Slp65 KO)	Fold Change (BtkSlp65 KO)	Fold Change (Rag1 KO)
*Genes known to be up-regulated in signaling-deficient pre-B cells*								
10463123	NM_009345	Dntt	N addition VDJ recombination	4.26E-08	8.46	16.99	22.49	39.19
10438064	NM_016982	Vpreb1	VpreB SLC component	7.58E-06	5.52	6.80	6.32	5.73
10438060	ENSMUST00000100136	Igll1	λ5 SLC component	2.17E-04	3.38	3.81	4.17	3.69
10427628	NM_008372	Il7r	IL-7 cytokine receptor	n.s.^c^	1.08	1.62	1.56	1.91
*Genes known to be down-regulated in signaling-deficient pre-B cells*								
10500677	NM_013486	Cd2	cell adhesion	2.57E-04	−4.82	−5.35	−20.52	−24.26
10469278	NM_008367	Il2ra	IL2 cytokine receptor CD25	1.60E-03	−5.21	−8.79	−15.90	−16.26
10450154	NM_010378	H2-Aa	MHC class II	1.45E-04	−2.04	−5.75	−13.16	−19.80
10562132	NM_001043317	Cd22	Siglec- family receptor	3.32E-05	1.29	1.14	−1.98	−6.06
*Transcription regulators and V(D)J recombination*								
10390640	NM_011771	Ikzf3	Aiolos DNA binding factor	7.05E-08	−1.66	−3.99	−29.34	−26.09
10384020	NM_017401	Polm	Polumerase mu	2.35E-06	−1.91	−4.17	−10.09	−12.25
10502510	NM_010723	Lmo4	DNA binding factor	1.70E-03	−1.90	−3.56	−5.70	−7.22
10404389	NM_013674	Irf4	DNA binding factor	7.87E-05	−1.60	−2.16	−4.75	−5.29
10438415	ENSMUST00000103752	Igl-V2	Ig V lambda light chain	6.90E-05	−3.41	−3.87	−4.57	−4.81
10438405	M94350	Igl-V1	Ig V lambda light chain	1.58E-06	−3.42	−3.07	−3.98	−6.20
10562812	NM_019866	Spib	Spi-B DNA binding factor	3.88E-04	−1.57	−1.94	−3.16	−3.22
10364559	NM_007880	Arid3a	Bright DNA binding factor	1.10E-03	−1.80	−2.16	−3.04	−3.18
10594001	NM_019689	Arid3b	DNA binding factor	5.74E-04	−1.79	−2.51	−2.91	−3.53
10554370	NM_175433	Zfp710	DNA binding factor	9.03E-03	−1.50	−1.64	−2.14	−2.99
10374333	NM_001025597	Ikzf1	Ikaros DNA binding factor	5.19E-05	−1.31	−1.77	−1.99	−2.44
10560964	NM_011138	Pou2f2	Oct-2 DNA binding factor	6.20E-03	−1.24	−1.30	−1.84	−2.56
10517090	NM_001080819	Arid1a	DNA binding factor	3.60E-03	−1.05	−1.25	−1.21	−2.16
10371662	NM_011461	Sp1c	Pu.1 Dna binding factor	n.s.	−1.04	−1.05	1.13	1.15
10585276	NM_011136	Pou2af1	OBF/OcaB DNA binding factor	n.s.	−1.10	−1.22	−1.22	−1.53
10359770	NM_011137	Pou2f1	DNA binding factor	n.s.	−1.10	−1.22	−1.22	−1.53
10370837	NM_011548	E2a	helix-loop-helix DNA binding factor	n.s.	−1.18	−1.15	−1.37	−1.68
10399691	NM_010496	Id2	inhibitor hlh DNA binding factor	n.s.	−1.39	−2.78	−3.18	−4.01
10509163	NM_008321	Id3	inhibitor hlh DNA binding factor	n.s.	1.46	1.39	1.29	−1.01
10576034	NM_008320	Irf8	DNA binding factor	n.s.	1.36	1.07	−1.07	−1.63
10512669	NM_008782	Pax5	DNA binding factor	n.s.	1.04	−1.22	−1.32	−1.92
10485372	NM_009019	Rag1	V(D)J recombination	n.s.	−1.45	−1.63	−2.03	−1.50
10485370	NM_009020	Rag2	V(D)J recombination	n.s.	−1.63	−1.21	−1.76	−1.72

a *p* value in ANOVA analysis.

b Fold change times up-regulated or down-regulated (−) when compared with WT (V_H_81X Tg *Rag1* ^−/−^) pre-B cells.

Groups are Rag1 KO *Rag1* ^−/−^ pro-B cells; Btk KO *Btk* ^−/−^ V_H_81X Tg *Rag1* ^−/−^ pre-B cells; Slp65 KO *Slp65* ^−/−^ V_H_81X Tg *Rag1* ^−/−^ pre-B cells; BtkSlp65 KO *Btk* ^−/−^ *Slp65* ^−/−^ V_H_81X Tg *Rag1^−/−^* pre-B cells.

c n.s., *p*>0.01.

Next, we used quantitative RT-PCR to confirm the observed differential expression of several TFs. Expression levels of these genes were indeed significantly reduced in a pre-BCR signaling-dependent manner, especially for Aiolos, Ikaros, and Irf4, with residual expression levels in *Btk* ^−/−^ *Slp65* ^−/−^ VH81x transgenic *Rag1* ^−/−^ pre-B cells that were ∼1%, ∼20%, and ∼9% of those observed in WT VH81x *Rag1* ^−/−^ mice, respectively (Figure 1C). In addition, we found moderate effects on *Obf1* (Oca-B) and *E2a* with residual expression levels of ∼28% and ∼44%, respectively. In chromatin immunoprecipitation (ChIP) assays, we observed in pre-B cells substantial binding of E2a protein to the intronic and 3′ κ enhancer regions and to the three V_κ_ regions analyzed. Under conditions of reduced pre-BCR signaling activity, E2a binding to the enhancers was essentially maintained (3′Eκ) or reduced (iEκ), but E2a binding to the V_κ_ regions was lost (Table S3). Consistent with the significant reduction of Ikaros expression in *Slp65* ^−/−^ pre-B cells, Ikaros binding to both κ enhancers and V_κ_ regions was undetectable in these cells (Table S3).

Taken together, from these findings we conclude that the five groups of pro-B/pre-B cells, representing a gradient of progressively diminished pre-BCR signaling, show in parallel a gradient of diminished modulation of many genes that signify pre-B cell differentiation, including key genes implicated in *Igκ* gene recombination.

### Progressively Diminished V_κ_ and J_κ_ GLTs in *Btk* ^−/−^, *Slp65* ^−/−^, and *Btk* ^−/−^ *Slp65* ^−/−^ Pre-B Cells

**Figure 2:**
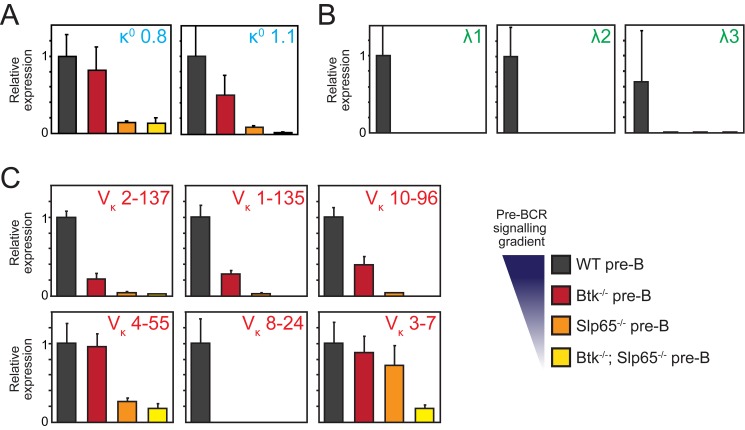
Reduction of Btk/Slp65-mediated pre-BCR signaling is associated with progressive loss of Igκ GLT. Quantitative RT-PCR analysis for κ^0^ (A), λ^0^ (B), and V_κ_ GLT (C) of FACS-sorted B220^+^CD19^+^ pre-B/pro-B cell fractions from the indicated mice on a VH81x transgenic *Rag1* ^−/−^ background. Expression levels were normalized to those of *Gapdh*, whereby the values in WT pre-B cells were set to one. Bars represent mean values and error bars denote standard deviations for four independent mice per group.

In these expression profiling studies, we only detected limited differences in germline transcription (GLT) over unrearranged J_κ_ and V_κ_ gene segments, which is thought to reflect locus accessibility [12]. However, we previously showed by serial-dilution RT-PCR that the levels of κ^0^ 0.8 and κ^0^ 1.1 germline transcripts, which are initiated in different regions 5′of J_κ_ and spliced to the C_κ_ region [49], are apparently normal in *Btk* ^−/−^ pre-B cells, modestly reduced in *Slp65* ^−/−^ pre-B cells, and severely reduced in *Btk* ^−/−^ *Slp65* ^−/−^ pre-B cells [34]. We could confirm these findings for κ^0^ GLT by quantitative RT-PCR assays on FACS-purified B220^+^CD19^+^ pro-B/pre-B cell fractions (Figure 2A). In agreement with our reported findings [34], we also found that *Btk* ^−/−^and *Slp65* ^−/−^ pre-B cells have defective λ^0^ transcription, which is initiated 5′ of the J_λ_ segments (Figure 2B) [49].

GLT across the V_κ_ region showed a similar pattern of sensitivity to pre-BCR signaling: decreased transcription of six individual V_κ_ regions tested (V_κ_3–7, V_κ_8–24, V_κ_4–55, V_κ_10–96, V_κ_1–35, and V_κ_2–137) correlated with decreased pre-BCR signaling activity (Figure 2C) in the pre-B cells of the four groups of mice. GLT over unrearranged V_λ_1 and V_λ_2 segments was strongly reduced in the absence of Btk or Slp65, as detected by the expression arrays (Table 1).

These observations indicate that *Igκ* locus accessibility, a hallmark of recombination-competent antigen receptor loci, is progressively reduced under conditions of diminishing pre-BCR signaling.

### Pre-BCR Signaling Induces Modulation of Long-Range Chromatin Interactions at the *Igκ* Locus

Accessibility of antigen receptor loci for V(D)J recombination is thought to be initiated by enhancers, in part through long-range chromatin interactions with promoters of noncoding transcription, resulting in the activation of germline transcription [8]. Because pre-BCR signaling affects the expression of GLT and various nuclear proteins that mediate long-range chromatin interactions and bind the κ enhancers, it is conceivable that pre-BCR signaling induces changes in the enhancer-mediated higher order chromatin structure of the *Igκ* locus that facilitates V_κ_ gene accessibility.

We therefore performed 3C-Seq analyses on FACS-purified B220^+^CD19^+^ fractions from the same five groups of mice (WT, *Btk* ^−/−^, *Slp65* ^−/−^, and *Btk* ^−/−^ *Slp65* ^−/−^ VH81x transgenic *Rag1* ^−/−^ pre-B cells, as well as *Rag1* ^−/−^ pro-B cells). Erythroid progenitors were analyzed in parallel as a nonlymphoid control, in which the *Igκ* locus was not contracted. Genome-wide chromatin interactions were measured for three regulatory elements involved in the control of *Igκ* locus accessibility and recombination: the iEκ and 3′Eκ enhancers [50]–[52] and the Sis element [20], which contain binding sites for Ikaros/Aiolos, E2a, and Irf4 [16],[17],[20],[38],[53].

**Figure 3:**
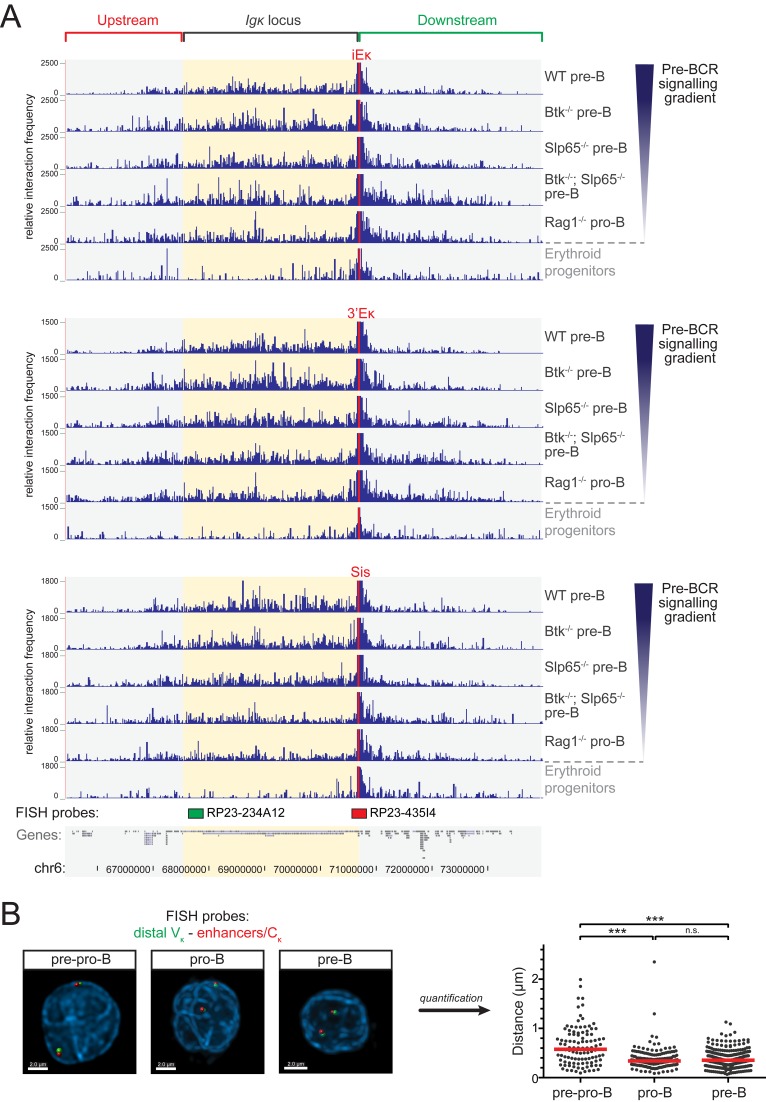
3C-Seq analysis of long-range chromatin interactions within the Igκ locus and flanking regions. (A) Overview of long-range interactions revealed by 3C-Seq experiments performed on the indicated cell fractions, representing a gradient of pre-BCR signaling, whereby the iEκ element (top), the 3′Eκ element (center), or the Sis element (bottom) was used as a viewpoint. Shown are the relative interaction frequencies (average of two replicate experiments) for the indicated genomic locations. The ∼8.4 Mb region containing the Ig*κ* locus (yellow shading) and flanking regions (cyan shading) is shown and genes and genomic coordinates are given (bottom). The locations of the two BAC probes used for 3D DNA-FISH are indicated by a green (distal V_κ_ probe) and red (proximal C_κ_/enhancer probe) rectangle (*bottom*). Pre-B cell fractions were FACS-purified from the indicated mice on a VH81x transgenic *Rag1* ^−/−^ background (see Figure 1 for gating strategy). Erythroid progenitor cells were used as a nonlymphoid control. (B) 3D DNA-FISH analysis comparing locus contraction in cultured bone-marrow–derived *E2a* ^−/−^ pre-pro-B, *Rag1* ^−/−^ pro-B, and VH81x *Rag1* ^−/−^ pre-B cells (see Figure S6 for phenotype of IL-7 cultured B-lineage cells). Locations of the BAC probes used are indicated at the bottom of panel A. Representative images for all three cell types are shown on the left, quantifications (>100 nuclei counted per cell type) on the right. The red lines indicate the median distance between the two probes. Statistical significance was determined using a Mann–Whitney U test (***p<0.001; n.s., not significant, *p*≥0.05).

In WT pre-B cells, all three regulatory elements showed extensive long-range chromatin interactions within the V_κ_ region and substantially less interactions with regions up- or downstream of the ∼3.2 Mb *Igκ* domain (Figure 3A; see Figure S2, Figure S3, and Figure S4 for line graphs), confirming previous observations [21]. Under conditions of reduced pre-BCR signaling activity, the three *Igκ* regulatory elements still showed strong interactions with the V_κ_ region. Surprisingly, even in the complete absence of pre-BCR signaling in *Rag1* ^−/−^ pro-B cells, long-range interactions were still observed at frequencies well above those seen in nonlymphoid cells, suggesting that a contracted *Igκ* locus topology is not strictly dependent on pre-BCR signaling (Figure 3A, Figure S2, Figure S3, and Figure S4). Next, we used 3D DNA FISH analyses using BAC probes hybridizing to the distal V_κ_ and C_κ_/enhancer regions to confirm that *Igκ* locus contraction was similar in *Rag-1* ^−/−^ pro-B cells and VH81x transgenic *Rag-1* ^−/−^ pre-B cells (both showing a contracted topology, compared with noncontracted pre–pro-B cells deficient for the TF E2a; Figure 3B).

Nevertheless, we did observe that pre-BCR signaling induced clear differences in interaction frequencies. Whereas an increase in pre-BCR signaling was associated with a decrease in the interaction frequencies between the two κ enhancers and regions flanking the *Igκ* locus (as also revealed by more detailed images of selected regions upstream and downstream of the *Igκ* domain; see Figure S5), the overall interaction frequency within the *Igκ* domain appeared unchanged (Figure S3, Figure S4, and Figure S5). Remarkably, interactions with the Sis element showed quite an opposite pattern: pre-BCR signaling correlated with increased overall interactions within the *Igκ* domain and did not substantially affect interaction frequencies in the *Igκ* flanking regions (Figure S2 and Figure S5).

Taken together, these analyses show that (i) the *Igκ* locus is already contracted at the pro-B cell stage and that (ii) pre-BCR signaling induces changes in long-range chromatin interactions, both within the *Igκ* locus and in the flanking regions.

### Pre-BCR Signaling Enhances Interactions of 3′Eκ and Sis, But Not iEκ, with V_κ_^+^ Fragments

**Figure 4:**
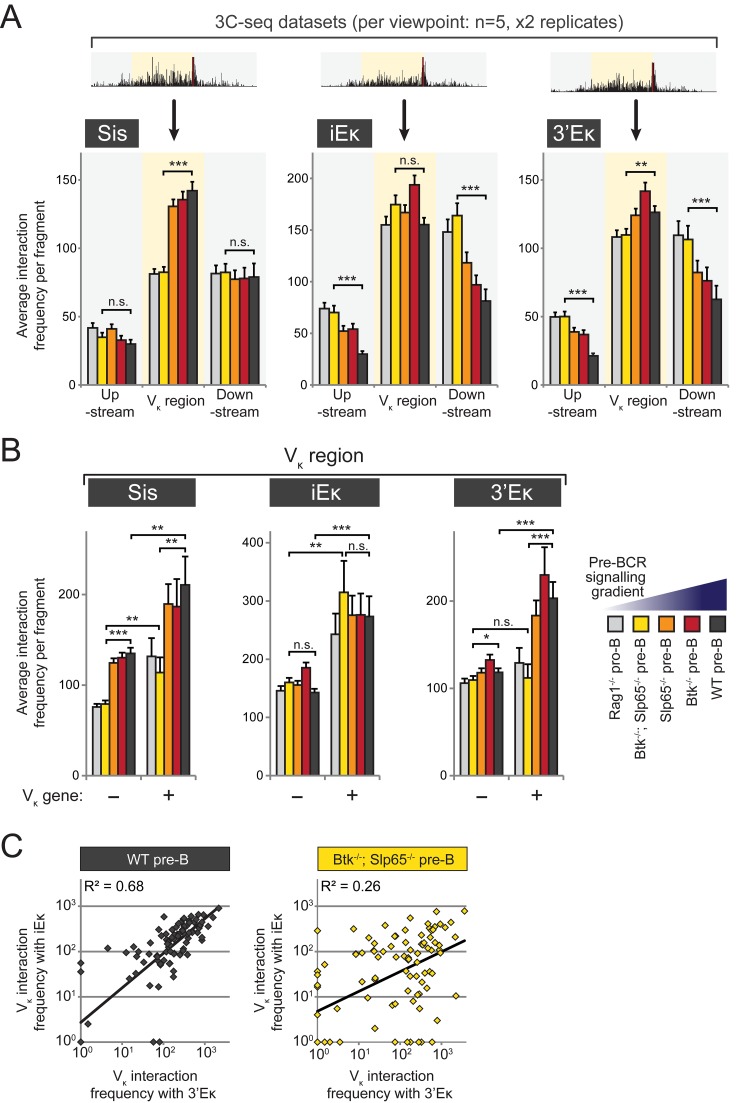
Modulation of long-range chromatin interactions within the Igκ locus by pre-BCR signaling. Quantitative analysis of 3C-Seq datasets using the three indicated κ regulatory elements as viewpoints. (A) Average long-range chromatin interaction frequencies (from two replicate 3C-seq experiments) with upstream (∼2.0 Mb), V_κ_ (∼3.2 Mb), and downstream (∼3.2 Mb) regions, as defined in Figure 3A, for the five B-cell precursor fractions representing a pre-BCR signaling gradient. Average interaction frequencies per region were calculated as the average number of 3C-Seq reads per restriction fragment within that region. See Materials and Methods section for more details. (B) Average interaction frequencies within the V_κ_ region were determined for fragments that do not (−) contain a functional V_κ_ gene and for those that do contain a functional V_κ_ gene (+). (C) Correlation plots of average interaction frequencies of the two enhancer elements with the 101 functional V_κ_ genes for WT pre-B cells (left) versus *Btk* ^−/−^ *Slp65* ^−/−^ pre-B cells (right). On the log scale, frequencies <1 were set to 10^0^. Statistical significance was determined using a Mann–Whitney U test (*p<0.05; **p<0.01; ***p<0.001; n.s., not significant, *p*≥0.05).

The differential effects of pre-BCR signaling on long-range chromatin interactions of the iEκ, 3′Eκ, and Sis elements clearly emerged in a quantitative analysis of the 3C-seq datasets (Figure 4A; see Materials and Methods for a detailed description of the quantification methods used). When pre-BCR signaling was absent (*Rag1* ^−/−^ pro-B cells) or very low (*Btk* ^−/−^ *Slp65* ^−/−^ pre-B cells), the average interaction frequencies were similar within the ∼3.2 Mb V_κ_ region and the ∼3.2 Mb downstream flanking region, for all three regulatory elements. Interaction frequencies with the upstream flanking region were lower, consistent with the larger chromosomal distance to the three viewpoints. The presence of increasing levels of Btk/Slp65-mediated pre-BCR signaling was associated with reduced interaction of iEκ and 3′Eκ with the *Igκ* flanking regions and with increased interaction of the Sis element and (to a lesser extent) 3′Eκ with the V_κ_ region (Figure 4A). As a result, for all three regulatory elements pre-BCR signaling resulted in a preference for interaction with fragments inside the V_κ_ region over fragments outside the V_κ_ region (Figure S7).

We next focused our analysis on the V_κ_ region and compared fragments that harbor a functional V_κ_ gene (V_κ_^+^ fragment) and those that do not (V_κ_^−^ fragment). When pre-BCR signaling was absent (*Rag1* ^−/−^ pro-B cells) or very low (*Btk* ^−/−^ *Slp65* ^−/−^ pre-B cells), the average interaction frequencies of the Sis or iEκ elements with V_κ_^+^ fragments were higher than with V_κ_^−^ fragments. The average interaction frequencies of 3′Eκ with V_κ_^+^ and V_κ_^−^ fragments, however, were similar (Figure 4B). Upon pre-BCR signaling, the Sis element showed an increase in interaction frequencies with both V_κ_^+^ and V_κ_^−^ fragments, with nevertheless an interaction preference for V_κ_^+^ fragments. In contrast, interaction frequencies between the iEκ element and V_κ_^+^ or V_κ_^−^ fragments were not modulated by pre-BCR signaling at all (Figure 4B). The 3′Eκ element exhibited yet another profile: pre-BCR signaling induced increased interaction frequencies specifically with V_κ_^+^ fragments, while interactions with V_κ_^−^ fragments were not notably modulated by pre-BCR signaling (Figure 4B). When we separately analyzed nonfunctional pseudo-V_κ_ genes, we found for the Sis and 3′Eκ elements that the interaction patterns with functional and nonfunctional V_κ_ genes were similar (Figure S8). In contrast, the iEκ enhancer did show an overall increased interaction frequency with V_κ_ functional genes, compared with nonfunctional V_κ_ genes, a phenomenon which was again independent from pre-BCR signaling (Figure S8).

The finding that interactions of V_κ_ genes with the intronic enhancer are already robust in pro-B cells, while those with the 3′κ enhancer are dependent on pre-BCR signaling, suggested that for individual V_κ_ genes pre-BCR signaling may result in more similar interaction frequencies with the two enhancers. To investigate this, we examined for all individual V_κ_ genes the correlation between their 3C-seq interaction frequencies with the iEκ and 3′κ elements and found that these were highly correlated in WT pre-B cells (R^2^ = 0.68; Figure 4C). Correlation was severely reduced when pre-BCR signaling was low in *Btk* ^−/−^ *Slp65* ^−/−^ pre-B cells (R^2^ = 0.26; Figure 4C). Similar pre-BCR signaling-dependent correlations were observed between V_κ_-interactions with the Sis element and those with the two enhancers (Figure S9). As the Sis element particularly suppresses recombination of the proximal V_κ_3 family, we investigated interaction correlations specifically for this V_κ_ family. Similar to our findings for all V_κ_ genes, a subanalysis showed strong correlations for the interactions of V_κ_3 family genes with iEκ, 3′κ, and Sis in WT pre-B cells, which were diminished when pre-BCR signaling was low, except for iEκ–Sis correlations, which were pre-BCR signaling-independent (Figure S9).

In summary, we conclude that pre-BCR signaling induces a redistribution of long-range interactions of the iEκ, 3′Eκ, and Sis elements, thereby restricting interactions towards the V_κ_ gene region. Moreover, upon pre-BCR signaling the long-range interactions mediated by 3′Eκ and Sis—but not those mediated by iEκ—become enriched for fragments harboring a V_κ_ gene, demonstrating increased proximity of 3′Eκ and Sis to V_κ_ genes. Finally, for individual V_κ_ genes, the interactions with iEκ, 3′Eκ, and Sis become highly correlated upon pre-BCR signaling, indicating that pre-BCR signals result in regulatory coordination between these three elements that govern *Igκ* locus recombination. In contrast, interactions between genes of the proximal V_κ_3 family, Sis and iEκ—but not 3′κ—appear to be coordinated already in the absence of pre-BCR signaling.

### Long-Range Chromatin Interactions of κ Regulatory Elements Correlate with V_κ_ Usage

**Figure 5:**
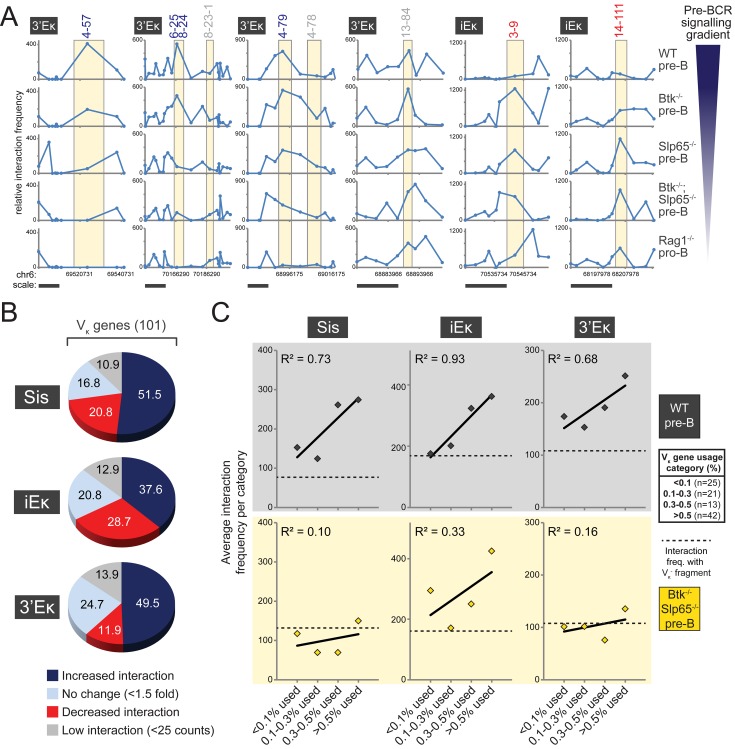
Long-range chromatin interactions of κ regulatory elements correlate with V_κ_ gene usage. (A) Selected examples of genomic regions containing V_κ_^+^ fragments, showing increased (V_κ_4–57, V_κ_6–25, V_κ_8–24, V_κ_4–79), stable (V_κ_8–23–1, V_κ_4–78, V_κ_13–84), or decreased (V_κ_3–9, V_κ_14–111) 3C-seq interaction frequencies with 3′Eκ or iEκ upon pre-BCR signaling. Averaged 3C-seq signals are plotted as a line graph, with the individual data points representing the center of the BglII restriction fragments. Yellow shading marks the BglII fragment on which the V_κ_ gene(s) is located. V_κ_ gene(s) are indicated (top) and chromosomal coordinates and scale bars (10 kb) are plotted (bottom). (B) Classification of V_κ_^+^ fragments, based on the effect of pre-BCR signaling on their interactions with the three κ regulatory elements indicated. Increase and decrease were defined as >1.5-fold change of interaction frequencies detected in WT pre-B cells versus *Btk* ^−/−^ *Slp65* ^−/−^ pre-B cells. (C) Correlation of average interaction frequencies (for the three κ regulatory elements indicated) with four V_κ_ usage categories ranging from low (<0.1%) to high usage (>0.5%, listed in the table on the right). Diamonds represent average interaction frequencies for *Btk* ^−/−^ *Slp65* ^−/−^ pre-B cells (yellow) and WT pre-B cells (grey). The dotted line in each graph depicts the average interaction frequency with fragments that do not contain a functional V_κ_ (V_κ_^−^). Primary V_κ_ gene usage data were taken from [54].

Next, we investigated the effects of pre-BCR signaling on the interaction frequencies of individual functional V_κ_ genes with the three κ regulatory elements (Figure 5A,B). The 3C-seq patterns of the majority (∼91%) of the 101 individual V_κ_^+^ fragments showed evidence for interaction with one or more of the κ regulatory elements (>25 average counts). When comparing *Btk* ^−/−^ *Slp65* ^−/−^ with WT pre-B cells, we observed that for a large proportion (∼38–52%) of V_κ_^+^ fragments, interaction frequencies increased upon pre-BCR signaling (Figure 5B). Smaller proportions of V_κ_^+^ fragments showed a decrease (∼12–29%) or were not significantly affected by pre-BCR signaling (∼17–25% with <1.5-fold change). The observed increase or decrease was not related to proximal or distal location of the V_κ_ genes, nor to their sense or antisense orientation (not shown). Distributions of the three different classes of V_κ_^+^ fragments showed substantial differences between the κ regulatory elements. For the Sis and 3′Eκ elements, more V_κ_^+^ fragments showed increased than decreased interactions (Figure 5B), in agreement with the signaling-dependent increase in average interaction frequencies of all V_κ_^+^ fragments (Figure 4B). In contrast, for the iEκ viewpoint, V_κ_^+^ fragments showing increased and decreased interactions were more equal in number, consistent with the limited effects of pre-BCR signaling on overall iEκ interaction frequencies of all V_κ_^+^ fragments (Figure 4B).

Although antigen receptor recombination is in principle regarded as a random process, a significant skewing of the primary Igκ repertoire of C57BL/6 mice was recently reported: one third of the V_κ_ genes was shown to account for >85% of the V_κ_ segments used by B cells [54]. To assess whether a correlation exists between usage of V_κ_ genes and their interaction frequencies with κ regulatory elements, we divided the V_κ_ genes into four usage categories (<0.1%, 0.1–0.3%, 0.3–0.5%, and >0.5%) and calculated their average 3C-Seq interaction frequencies with Sis, iEκ, and 3′κ (Figure 5C). In WT pre-B cells, V_κ_ usage showed a strong positive correlation with 3C-Seq interaction frequencies for all three regulatory elements (R^2^ = ∼0.7–0.9; Figure 5C). These correlations were pre-BCR signaling-dependent, since in *Btk* ^−/−^ *Slp65* ^−/−^ pre-B cells, they were reduced (for iEκ; R^2^ = 0.33) or absent (for Sis and 3′κ; R^2^<0.10 and R^2^<0.16, respectively) (Figure 5C).

Collectively, our results indicate that specifically the most frequently used V_κ_ genes are the main interaction targets of κ regulatory elements, whereby pre-BCR signaling completely underlies this specificity for the Sis and 3′Eκ elements, and to a lesser extent for iEκ.

### Long-Range Interactions with κ Regulatory Elements Correlate with the Presence of Ctcf, Ikaros, E2a, and H3K4 Hypermethylation

Next, we investigated whether long-range interactions between κ regulatory elements and the V_κ_ region correlated with the presence of the TFs Ctcf [21], Ikaros [55], and E2a [56], which have been implicated in *Igκ* locus recombination [21],[37],[55],[57],[58]. Notably, Ikaros and E2a both strongly bind all three κ regulatory elements, while the Sis element is also occupied by Ctcf ([21]; unpublished data).

**Figure 6:**
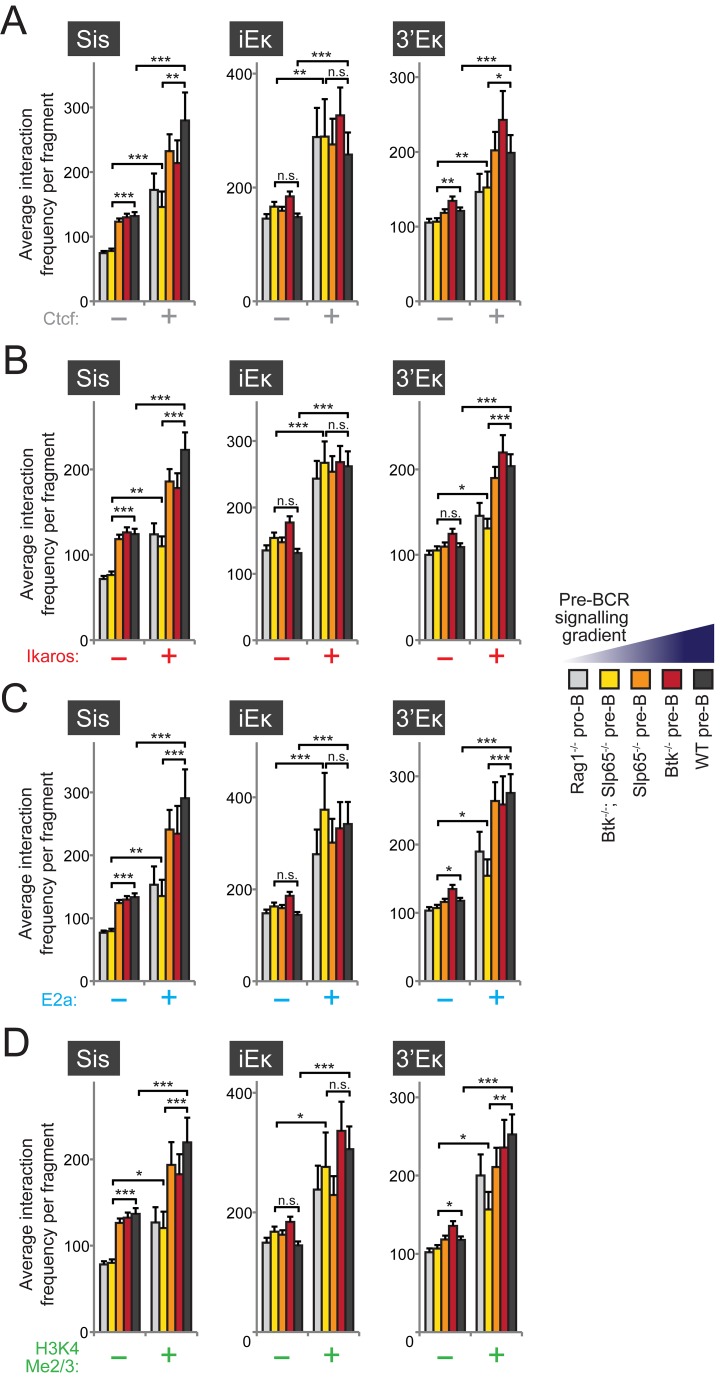
Long-range chromatin interactions of κ regulatory elements correlate with TF binding and histone modifications. (A-D) For fragments within the V_κ_ region, average 3C-seq interaction frequencies were calculated for fragments that did (+) or did not (−) contain binding sites for TFs or H3K4 histone modifications (as determined by previous ChIP-Seq studies; see Materials and Methods for references). Data for the three viewpoint and the five B-cell precursor fractions representing a pre-BCR signaling gradient are shown for Ctcf (A), Ikaros (B), E2a (C), and H3K4 di- and tri-methylation (Me2/3). Statistical significance was determined using a Mann–Whitney U test (*p<0.05; **p<0.01; ***p<0.001; n.s., not significant, *p*≥0.05).

**Figure 7:**
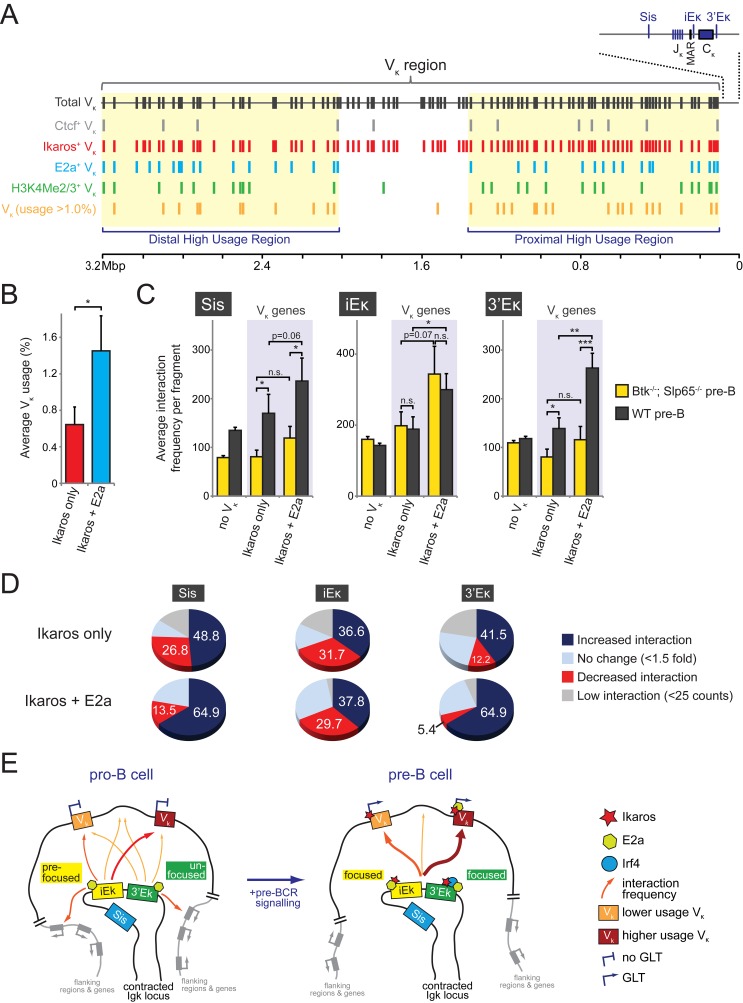
Proximity of V_κ_ genes to E2a binding sites correlates with frequencies of long-range interactions. (A) Schematic representation of the *Igκ* locus, showing the location of all functional V_κ_ (grey, top), J_κ_ and C_κ_ gene segments, and the κ regulatory elements Sis, iEκ, and 3′Eκ. MAR, matrix attachment region. V_κ_ genes within close proximity (as defined by colocalization on the same 3C-Seq restriction fragment) to the indicated TFs or H3K4 hypermethylation (as detected by previous ChIP-seq studies; see Materials and Methods for references) are shown. At the bottom, highly used (>1.0% used) V_κ_ gene segments are depicted (orange), which cluster within two large high-usage domains (yellow shading). Primary V_κ_ gene usage data was taken from [54]. (B) Average usage of V_κ_ genes marked only by an Ikaros binding site or those marked by binding sites of both Ikaros and E2a. (C) Comparison of average interaction frequencies (for the three κ regulatory elements indicated) between V_κ_^−^ fragments (no V_κ_), V_κ_^+^ fragments containing an Ikaros binding site only, and V_κ_^+^ fragments containing both an Ikaros and E2a binding site. Bars represent average frequencies for *Btk* ^−/−^ *Slp65* ^−/−^ pre-B cells (yellow) and WT pre-B cells (grey). (D) Classification of V_κ_^+^ fragments, containing an Ikaros binding site only (top) or containing both an Ikaros and E2a binding site (bottom), based on the effect of pre-BCR signaling on their interactions with the three κ regulatory elements indicated. Increase and decrease were defined as >1.5-fold change of interaction frequencies detected in WT pre-B cells versus *Btk* ^−/−^ *Slp65* ^−/−^ pre-B cells. (E) Proposed model of pre-BCR signaling-mediated changes in κ enhancer action. In pro-B cells (left) the enhancers show minimal coordination and their interactions are not yet (fully) focused on the V_κ_ genes. Upon pre-BCR signaling and differentiation to pre-B cells (right), TFs bind the locus to coordinate enhancer action and focus their interactions to the V_κ_ genes, inducing germline transcription (GLT) and accessibility to the V(D)J recombinase. See Discussion for more details. Statistical significance was determined using a Mann–Whitney U test (*p<0.05; **p<0.01; ***p<0.001; n.s., not significant, *p*≥0.05).

Remarkably, we found similar striking correlations between the presence of *in vivo* binding sites for each of these TFs (as determined by ChIP experiments; see Materials and Methods for the relevant references) and long-range chromatin interactions with the κ regulatory elements (Figure 6A–C), even though Ctcf sites are mostly located in between V_κ_ genes [21] and Ikaros/E2a sites were frequently found close to V_κ_ gene promoter regions ([2]; Figure 7A). Even when pre-BCR signaling was absent (*Rag1* ^−/−^ pro B cells) or very low (*Btk* ^−/−^ *Slp65* ^−/−^ pre-B cells), the average interaction frequencies of the κ regulatory elements with fragments containing Ctcf, Ikaros, or E2a bindings sites were higher than those without binding sites. Irrespective of the presence or absence of bindings sites for these TFs, we found that upon pre-BCR signaling interaction frequencies with the Sis element increased and those with the iEκ did not change. In contrast, for the 3′Eκ we found that pre-BCR signaling specifically increased interaction frequencies with fragments occupied by Ctcf, Ikaros, or E2a.

Finally, we found that the presence of di- or trimethylation of histone 3 lysine 4 (H3K4Me2/3), an epigenetic signature associated with locus accessibility [59] and Rag-binding [60],[61], also correlated with increased interaction frequencies with κ regulatory elements, revealing a similar pre-BCR signaling dependency as seen for the TFs analyzed (Figure 6D).

We conclude that the presence of essential TFs or H3K4Me2/3 in the V_κ_ region strongly correlates with the formation of long-range chromatin interactions with the κ regulatory elements, and that for the Sis and 3′Eκ elements this interaction preference is further enhanced by pre-BCR signaling.

### Proximity of V_κ_ Genes to E2a Binding Sites Correlates with High V_κ_ Usage and Increased Long-Range Chromatin Interactions

Since the long-range interactions with κ regulatory elements correlated with the presence of TFs implicated in *Igκ* recombination, we next asked whether the κ regulatory elements preferentially interacted with V_κ_ genes that are in close proximity to binding sites for Ctcf, Ikaros, or E2a.

Strikingly, the majority of functional V_κ_ genes (95/101) was found to have an Ikaros binding site in close proximity—that is, located on the same 3C-seq restriction fragment (average length of ∼3 kb, unpublished data) (Figure 7A). Proximity of V_κ_ genes to an E2a binding site (37%) or H3K4Me2/3 positive region (∼28%) is more selective, while only a small fraction of V_κ_ genes are close to Ctcf binding sites (∼12%) ([22]; Figure 7A). All V_κ_ genes marked by E2a, Ctcf, H3K4Me2/3, or a combination of these also contain an Ikaros binding site. Frequently used V_κ_ genes (>1.0% usage; 33/101 genes) were located in two separate regions, a proximal and a distal region, which also contained virtually all E2a and H2K4Me2/3-marked V_κ_ genes (Figure 7A).

We found that V_κ_ genes marked by both Ikaros and E2a were used substantially more often than those only bound by Ikaros (Figure 7B), suggesting that these V_κ_ genes are preferentially targeted for V_κ_-to-J_κ_ gene rearrangement. Our 3C-seq analyses showed that in WT pre-B cells, interaction frequencies with the three κ regulatory elements were higher for Ikaros/E2a-marked V_κ_ genes compared to genes marked by Ikaros binding alone (Figure 7C). In fact, V_κ_^+^ restriction fragments containing an Ikaros binding site but not an E2a binding site showed interaction frequencies similar to V_κ_^−^ restriction fragments. Under conditions of very low pre-BCR signaling (in *Btk* ^−/−^ *Slp65* ^−/−^ pre-B cells), we observed strongly reduced interaction frequencies of V_κ_^+^ E2a binding restriction fragments with the Sis and 3′Eκ elements. These interaction frequencies were in the same range as those of V_κ_^−^ fragments or V_κ_^+^ fragments that harbored an Ikaros site only (Figure 7C). Interaction frequencies with the iEκ enhancer, however, were independent of pre-BCR signaling. As shown in Figure 7D, for the majority of Ikaros/E2a-marked V_κ_^+^ fragments (65%), pre-BCR signaling was associated with increased interactions with the Sis and 3′Eκ elements (comparing wild-type and *Btk* ^−/−^ *Slp65* ^−/−^ pre-B cells). In these analyses, only ∼13.5% and ∼5.4% of Ikaros/E2a-marked V_κ_^+^ fragments showed a decreased interaction frequency upon pre-BCR signaling. In contrast, almost equal proportions of Ikaros/E2a-marked V_κ_^+^ fragments showed increased (∼37%) and decreased (∼30%) interactions with iEκ upon pre-BCR signaling.

Taken together, these data reveal strong positive correlations between the presence of E2a binding sites, V_κ_ usage, and long-range chromatin interactions with κ regulatory elements in pre-B cells. Remarkably, for the iEκ element, these correlations are largely independent of Btk/Slp65-mediated pre-BCR signaling, whereas for the 3′Eκ they are completely dependent on signaling.

## Discussion

During B-cell development the pre-BCR checkpoint is known to regulate the expression of many genes, part of which control the increase in *Igκ* locus accessibility to the V(D)J recombinase complex. However, it remained unknown how pre-BCR signaling events affect accessibility in terms of *Igκ* locus contraction and topology.

Here we identified numerous genes involved in *IgL* chain recombination, chromatin modification, signaling, and cell survival to be aberrantly expressed in pre-B cells lacking the pre-BCR signaling molecules Btk and/or Slp65. We found that GLT over the Vκ region, reflecting Vκ accessibility, is strongly reduced in these cells. We used 3C-Seq to show that in pro-B cells both the intronic and the 3′ κ enhancers frequently interact with the ∼3.2 Mb Vκ region, as well as with *Igκ* flanking sequences, indicating that the *Igκ* locus is already contracted at the pro-B cell stage. 3C-Seq analyses in wild-type and Btk/Slp65 single- and double-deficient pre-B cells demonstrated that pre-BCR signaling significantly affects *Igκ* locus topology. First, pre-BCR signaling reduces the interactions of the intronic and 3′κ enhancers with *Igκ* flanking regions, effectively focusing enhancer action towards the V_κ_ region to facilitate V_κ_-to-J_κ_ recombination. Second, pre-BCR signaling strongly increases nuclear proximity of the 3′κ enhancer to V_κ_ genes, whereby this increase is more substantial for more frequently used V_κ_ genes and for V_κ_ genes close to a binding site for the basic helix-loop-helix protein E2a. Third, pre-BCR signaling augments interactions between κ regulatory elements and fragments within the V_κ_ region bound by the key B-cell TFs Ikaros and E2a and the architectural protein Ctcf. Fourth, pre-BCR signaling has limited effects on interactions of the intronic κ enhancer with fragments within the *Igκ* locus, as this enhancer already displays interaction specificity for functional V_κ_ genes and TF-bound regions in pro-B cells. Fifth, pre-BCR signaling has limited effects on the interactions between the intronic or 3′κ enhancers and fragments that do not contain a V_κ_ gene or an Ikaros, E2a, or Ctcf binding site, emphasizing the specificity of pre-BCR signaling-induced changes in *Igκ* locus topology. Sixth, pre-BCR signaling appears to induce mutual regulatory coordination between the three regulatory elements, as their interaction profiles with individual V_κ_ genes become highly correlated upon signaling. Finally, pre-BCR signaling increases interactions of the Sis element with DNA fragments in the *Igκ* locus, irrespective of the presence of a V_κ_ gene or TF. Collectively, our findings demonstrate that pre-BCR signals relayed through Btk and Slp65 are required to create a chromatin environment that facilitates proper *Igκ* locus recombination. This multistep process is initiated by up-regulation of key TFs like Aiolos, Ikaros, Irf4, and E2a. These proteins are then recruited to or further accumulate at the *Igκ* locus and its regulatory elements, resulting in a specific fine-tuning of enhancer-mediated locus topology that increases locus accessibility to the Rag recombinase proteins.

Importantly, the presence of strong lineage-specific interaction signals between the C_κ_/enhancer region and distal V_κ_ genes in pro-B cells indicates that the *Igκ* locus is already contracted at this stage. In contrast to a previous microscopy study indicating that *Igκ* locus contraction did not occur until the small pre-B cell stage [36], our 3D DNA FISH analysis indeed detected similar nuclear distances between distal V_κ_ and the C_κ_/enhancer region in cultured pro-B and pre-B cells. Recently Hi-C was employed to study global early B cell genomic organization whereby substantial interaction frequencies were found between the intronic κ enhancer and the V_κ_ region in pro-B cells [40]. E2a-deficient pre–pro-B cells, which are not yet fully committed to the B-cell lineage [62], showed very few interactions among the iEκ and the distal part of the V_κ_ region [40], resembling the interactions we observed in nonlymphoid cells (Figure 3A). Accordingly, 3D-FISH analysis showed that the *Igκ* locus adopted a noncontracted topology in these pre–pro-B cells (Figure 3B). These data indicate that *Igκ* locus contraction is already achieved in pro-B cells and depends on the presence of E2a. Supporting this notion, active histone modifications and E2a were already detected at the κ enhancers and V_κ_ genes at the pro-B cell stage [56],[63], whereby E2a was frequently found at the base of long-range chromatin interactions together with Ctcf and Pu.1, possibly acting as “anchors” to organize genome topology [40]. The observed correlation between E2a binding, V_κ_ gene usage and iEκ proximity in pro-B cells (Figure 5C, Figure 7C) further strengthens an early critical role for E2a in regulating *Igκ* locus topology, V_κ_ gene accessibility, and recombination.

Our 3C-seq experiments revealed that pre-BCR signaling is not required to induce long-range interactions between the κ regulatory elements and distal parts of the V_κ_ locus, indicating that TFs strongly induced by signaling—that is, Aiolos, Ikaros, and Irf4—are not strictly necessary to form a contracted *Igκ* locus. Prime candidates for achieving Igκ locus contraction at the pro-B cell stage are E2a and Ctcf, as they have been implicated in regulating Ig locus topology [21],[40],[64],[65] and E2a already marks frequently used V_κ_ genes at the pro-B cell stage (Figure 7), although we did observe reduced E2a expression and binding to the iEκ enhancer and V_κ_ genes when pre-B cell signaling was low (Figure 1 and Table S3), suggesting that pre-BCR signaling is required for high-level E2a occupancy of the V_κ_ genes. We previously reported that *Igκ* gene recombination can occur in the absence of Ctcf and that Ctcf mainly functions to limit interactions of the κ enhancers with proximal V_κ_ regions and to prevent inappropriate interactions between these strong enhancers and elements outside the Igκ locus [21]. Because at the pro-to-pre–B cell transition Aiolos, Ikaros, and Irf4 are recruited to the *Igκ* locus and histone acetylation and H3K4 methylation increases [17],[38],[63],[66], we hypothesize that pre-BCR–induced TFs act upon an E2a/Ctcf-mediated topological scaffold to further refine the long-range chromatin interactions of the κ regulatory elements. Hereby, these TFs mainly act to focus and to coordinate the interactions of the two κ enhancers to the V_κ_ gene segments, in particular to frequently used V_κ_ genes, thereby increasing their accessibility for recombination (see Figure 7E for a model of pre-BCR signaling-induced changes in Igκ locus accessibility).

In this context, our 3C-seq data show that the two κ enhancer elements have distinct roles. Both 3′Eκ and iEκ elements manifest interaction specificity for highly used, E2a-marked, V_κ_ genes. However, whereas iEκ already shows this specificity in pro-B cells (although pre-BCR signaling does augment this specificity), 3′Eκ only does so in pre-B cells upon pre-BCR signaling. These observations indicate that iEκ is already “prefocused” at the pro-B cell stage and that pre-BCR signals are required to fully activate and focus the 3′Eκ to allow synergistic promotion of *Igκ* recombination by both enhancers (see Figure 7E) [52]. In agreement with such distinct sequential roles, iEκ and not the 3′Eκ was found to be required for the initial increase in *Igκ locus* accessibility, which occurred upon binding of E2a only [37],[38],[67]. The 3′Eκ on the other hand requires binding of pre-BCR signaling-induced Irf4 to promote locus accessibility [19],[38], followed by further recruitment of E2a to both κ enhancers and highly used V_κ_ genes (Table S3 and [38],[57]).

The Sis regulatory element was shown to dampen proximal V_κ_–J_κ_ rearrangements and to specify the targeting of *Igκ* transgenes to centromeric heterochromatin in pre-B cells [20]. As Sis is extensively occupied by the architectural Ctcf protein and deletion of Sis or Ctcf both resulted in increased proximal V_κ_ usage [21],[23], it was postulated that Sis functions as a barrier element to prevent the *κ* enhancers from too frequently targeting proximal V_κ_ genes for recombination. In this context, we now provide evidence that interactions between the proximal V_κ_ genes, Sis, and iEκ—but not 3′κ—are already coordinated before pre-BCR signaling occurs (Figure S9). Perhaps not surprisingly, Sis-mediated long-range chromatin interactions displayed a pattern and pre-BCR signaling response that was different from the κ enhancers. Unlike for the enhancers, upon pre-BCR signaling, Sis-mediated interactions with regions outside the *Igκ* locus were maintained and interaction within the V_κ_ region increased, irrespective of the presence of V_κ_ genes or TF binding sites. Because Sis is involved in targeting the nonrecombining *Igκ* allele to heterochromatin [20], the observed interaction pattern of the Sis element might reflect its action in pre-B cells to sequester the nonrecombining *Igκ* locus and target it towards heterochromatin. This might also explain the increased interaction frequencies of Sis with highly used V_κ_ genes upon pre-BCR signaling (Figures 5C and 7C), as such highly accessible genes likely require an even tighter association with Sis and heterochromatin to prevent undue recombination.

Surprisingly, we observed a striking correlation between Ikaros binding and V_κ_ gene location (94% of V_κ_ genes were in close proximity to an Ikaros binding site; Figure 7A). Although Ikaros and Aiolos have a positive role in regulating gene expression during B-cell development [55],[58] and Ikaros is required for *IgH* and *IgL* recombination [39],[58], Ikaros has also been reported to silence gene expression through its association with pericentromeric heterochromatin [68] or through recruitment of repressive cofactor complexes [69],[70]. Recruitment of Ikaros to the *Igκ* locus was found increased in pre-B cells as compared to pro-B cells [63], in agreement with its up-regulation in pre-B cells (Figure 1). Furthermore, Ikaros binds the Sis element, where it was suggested to mediate heterochromatin targeting of *Igκ* alleles by the Sis region [20]. Aiolos, although not essential for B-cell development like Ikaros [58],[71], is strongly induced by pre-B cell signaling and has been reported to cooperate with Ikaros in regulation gene expression [27]. Although their synergistic role during *IgL* chain recombination has not been extensively studied, the Ikaros/Aiolos ratio changes upon pre-BCR signaling (Figure 1). Increased recruitment of Ikaros/Aiolos to V_κ_ genes and the κ enhancers likely increases Igκ locus accessibility and contraction (see Figure 6), as Ikaros was very recently shown to be essential for *IgL* recombination [58]. On the other hand, it is conceivable that on the nonrecombining allele, increased recruitment of Ikaros/Aiolos to V_κ_ genes and the Sis region could facilitate silencing of this allele. Further investigations using allele-specific approaches [72] will be required to clarify the allele-specific action of the Sis element during *Igκ* recombination.

In summary, by investigating the effects of a pre-BCR signaling gradient—rather than deleting individual TFs—we have taken a more integrative approach to study the regulation of *Igκ* locus topology. Our 3C-Seq analyses in wild-type, Btk, and Slp65 single- and double-deficient pre-B cells show that interaction frequencies between Sis, iEκ, or 3′ Eκ and the V_κ_ region are already high in pro-B cells and that pre-BCR signaling induces accessibility through a functional redistribution of long-range chromatin interactions within the V_κ_ region, whereby the iEκ and 3′Eκ enhancer elements play distinct roles.

## Materials and Methods

### Mice

VH81x transgenic mice [73] on the *Rag-1* ^−/−^ background [74] that were either wild-type, *Btk* ^−/−^ [75], *Slp65* ^−/−^ [42], or *Btk* ^−/−^ *Slp65* ^−/−^ have been previously described [34]. Mice were crossed on the C57BL/6 background for >8 generations, bred, and maintained in the Erasmus MC animal care facility under specific pathogen-free conditions and were used at 6–13 wk of age. Experimental procedures were reviewed and approved by the Erasmus University Committee of Animal Experiments.

### Flow Cytometry

Preparation of single-cell suspensions and incubations with monoclonal antibodies (mAbs) were performed using standard procedures. Bone marrow B-lineage cells were purified using fluorescein isothiocyanate (FITC)-conjugated anti-B220(RA3-6B2) and peridinin chlorophyll protein (PCP)-conjugated anti-CD19, together with biotinylated mAbs specific for lineage markers Gr-1, Ter119, and CD11b and APC-conjugated streptavidin as a second step to further exclude non-B cells. Cells were sorted with a FACSARia (BD Biosciences). The following mAbs were used for flow cytometry: FITC-, PerCP–anti-B220 (RA3-6B2), phycoerythrin (PE)–anti-CD2 (LFA-2), PCP-, allophycocyanin (APC)- or APC–Cy7–anti-CD19 (ID3), PE-, or APC anti-CD43 (S7). All these antibodies were purchased from BD Biosciences or eBiosciences. Samples were acquired on an LSRII flow cytometer (BD Biosciences) and analyzed with FlowJo (Tree Star) and FACSDiva (BD Biosciences) software.

### Quantitative RT-PCR and DNA Microarray Analysis

Extraction of total RNA, reverse-transcription procedures, design of primers, and cDNA amplification have been described previously [21]. Gene expression was analyzed using an ABI Prism 7300 Sequence Detector and ABI Prism Sequence Detection Software version 1.4 (Applied Biosystems). All PCR primers used for quantitative RT-PCR of TFs or κ^0^, λ^0^, and V_κ_ GLT are described in [21], except for Obf1 (forward 5′-CCTGGCCACCTACAGCAC-3′, reverse 5′-GTGGAAGCAGAAA CCTCCAT-3′, obtained from the Roche Universal Probe Library).

Biotin-labeled cRNA was hybridized to the Mouse Gene 1.0 ST Array according to the manufacturer's instructions (Affymetrix); data were analyzed with BRB-ArrayTools (version 3.7.0, National Cancer Institute) using Affymetrix CEL files obtained from GCOS (Affymetrix). The RMA approach was used for normalization. The TIGR MultiExperiment Viewer software package (MeV version 4.8.1) was used to perform data analysis and visualize results [45]. One-way ANOVA analysis of the five experimental groups of B cells was used to identify genes significantly different from wild-type VH81X Tg *Rag1* ^−/−^ pre-B cells (*p*<0.01).

### Chromatin Immunoprecipitation (ChIP)

ChIP experiments were performed as previously described [76] using FACS sorted bone marrow pre-B cell fractions (0.3–2.0 million cells per ChIP). Antibodies against E2a (sc-349, Santa Cruz Biotechnology) and Ikaros (sc-9861, Santa Cruz Biotechnology) were used for immunoprecipitation. Purified DNA was analyzed by quantitative RT-PCR as described above. Primer sequences are available on request.

### Chromosome Conformation Capture Coupled to High-Throughput Sequencing (3C-Seq)

3C-Seq experiments were essentially carried out as described previously [21],[41]. For 3C-Seq library preparation, BglII was used as the primary restriction enzyme and NlaIII as a secondary restriction enzyme. 3C-seq template was prepared from WT E13.5 fetal liver erythroid progenitors and FACS-sorted bone marrow pro-B cell or pre-B cell fractions (see above) from pools of 4–6 mice. In total, between 1 and 8 million cells were used for 3C-seq analysis. Primers for the Sis, iEκ, and 3′Eκ viewpoint-specific inverse PCR were described previously [21]. 3C-seq libraries were sequenced on an Illumina Hi-Seq 2000 platform. 3C-Seq data processing was performed as described elsewhere [41],[77]. Two replicate experiments were sequenced for each genotype and viewpoint, and normalized interaction frequencies per BglII restriction fragment were averaged between the two experiments.

For quantitative analysis, the *Igκ* locus and surrounding sequences were divided into three parts (mm9 genome build): a ∼2 Mb upstream region (chr6:65,441,978–67,443,029; 759 fragments), a ∼3.2 Mb V_κ_ region (chr6:67,443,034–70,801,754; 1,290 fragments) and a downstream ∼3.2 Mb region (chr6:70,801,759–73,993,074; 1,143 fragments). For each cell type (as described above) sequence read counts within individual BglII restriction fragments were normalized for differences in library size (expressed as “reads per million”; see [74]) and averaged between the two replicates before further use in the various calculations. Very small BglII fragments (<100 bp) were excluded from the analysis. Fragments in the immediate vicinity of the regulatory elements (chr6:70,659,392–70,693,183; 10 fragments) were also excluded because of high levels of noise around the viewpoint, a characteristic of all 3C-based experiments. V_κ_ gene coordinates (both functional genes and pseudogenes) were obtained from IMGT [11] and NCBI (Gene ID: 243469) databases. V_κ_ gene usage data (C57BL/6 strain, bone marrow) were obtained from [54]. ChIP-seq datasets were obtained from [21] (Ctcf), [55] (Ikaros), and [56] (E2a, H3K4Me2, and H3K4Me3). V_κ_ genes were scored positive for TF binding sites or for a histone modification, if they were located on the same BglII restriction fragment (corresponding to the 3C-Seq analysis).

### 3D DNA Immuno-FISH

*Rag-1* ^−/−^ pro-B and *Rag-1* ^−/−^;VH81X pre-B cells were isolated from femoral bone marrow suspensions by positive enrichment of CD19^+^ cells using magnetic separation (Miltenyi Biotec). Cells were cultured for 2 wk in Iscove's Modified Dulbecco's medium containing 10% fetal calf serum, 200 U/ml penicillin, 200 mg/ml streptomycin, 4 nM L-glutamine, and 50 µM β-mercaptoethanol, supplemented with IL-7 and stem cell factor at 2 ng/ml. *E2a* ^−/−^ hematopoietic progenitors were grown as described previously [78]. Prior to 3D-FISH analysis, cells were characterized by flow cytometric analysis of CD43, CD19, and CD2 surface marker expression to verify their phenotype (Figure S6).

3D DNA FISH was performed as described previously [79] with BAC clones RP23-234A12 and RP23-435I4 (located at the distal end of the V_κ_ region and at the C_κ_/enhancer region, respectively; Figure 3A) obtained from BACPAC Resources (Oakland, CA). Probes were directly labeled with Chromatide Alexa Fluor 488-5 dUTP and Chromatide Alexa Fluor 568-5 dUTP (Invitrogen) using Nick Translation Mix (Roche Diagnostics GmbH).

Cultured primary cells were fixed in 4% paraformaldehyde, and permeabilized in a PBS/0.1% Triton X-100/0.1% saponin solution and subjected to liquid nitrogen immersion following incubation in PBS with 20% glycerol. The nuclear membranes were permeabilized in PBS/0.5% Triton X-100/0.5% saponin prior to hybridization with the DNA probe cocktail. Coverslips were sealed and incubated for 48 h at 37°C, washed, and mounted on slides with 10 µl of Prolong gold anti-fade reagent (Invitrogen).

Pictures were captured with a Leica SP5 confocal microscope (Leica Microsystems). Using a 63× lens (NA 1.4), we acquired images of ∼70 serial optical sections spaced by 0.15 µm. The datasets were deconvolved and analyzed with Huygens Professional software (Scientific Volume Imaging, Hilversum, the Netherlands). The 3D coordinates of the center of mass of each probe were transferred to Microsoft Excel, and the distances separating each probe were calculated using the equation: √(Xa−Xb)2+(Ya−Yb)2+(Za−Zb)2, where X, Y, and Z are the coordinates of object a or b.

### Statistical Analysis

Statistical significance was analyzed using a nonparametric Mann–Whitney U test (IBM SPSS Statistics 20). The *p* values<0.05 were considered significant.

### Accession Numbers

3C-seq and microarray expression datasets have been submitted to the Sequence Read Archive (SRA, accession number SRP032509) and Gene Expression Omnibus (GEO, accession number GSE53896), respectively.

## Supporting Information

Figure S1 **Gene distance matrix analysis using gene expression profiling data from pre-B/pro-B cell fractions representing a pre-BCR signaling gradient.** Microarray expression profiling was performed on three or four independent FACS-purified B220^+^CD19^+^ pre-B cell fractions from wild-type (WT), *Btk*, and *Slp65* single- and double-deficient VH81x transgenic *Rag1* ^−/−^ mice (see Figure 1 for gating strategy). The TIGR Multi Experiment Viewer software package (MeV version 4.8.1) was used to perform a one-way ANOVA analysis (*p*<0.01) and identify genes differentially expressed within the five B-cell fractions (versus VH81X Tg *Rag1* ^−/−^ pre-B cells). The software was subsequently used to create a gene distance matrix of highly significant genes, resulting in the depicted plot. Differences in gene expression profiles are depicted as a color code; darker colors indicate greater similarity, and brighter colors less similarity between groups. Consistent with the unsupervised clustering analysis shown in Figure 1B, a clear gene expression gradient among the five B cell groups emerges in which *Btk* ^−/−^ *Slp65* ^−/−^ pre-B and *Rag1* ^−/−^ pro-B cells show highly comparably expression signatures.
(TIF)

Figure S2 **Locus-wide 3C-Seq analysis of the** ***Ig*** **κ region (Sis viewpoint) plotted as line graphs.** Overview of long-range interactions revealed by 3C-Seq experiments performed on the indicated cell fractions, representing a gradient of pre-BCR signaling. Shown are the relative interaction frequencies (average of two replicate experiments) for the Sis viewpoint per 100 kb region across the *Ig*κ locus, plotted as line graphs. The bottom graph shows an overlay of the WT and *Btk* ^−/−^ *Slp65* ^−/−^ pre-B cell interaction frequencies. The ∼8.4 Mb region containing the *Ig*κ locus (yellow shading) and flanking regions (cyan shading) is depicted. Pre-B cell fractions were FACS-purified from the indicated mice on a VH81x transgenic *Rag1* ^−/−^ background (see Figure 1 for gating strategy).
(TIF)

Figure S3 **Locus-wide 3C-Seq analysis of the** ***Ig*** **κ region (iEκ viewpoint) plotted as line graphs.** Overview of long-range interactions revealed by 3C-Seq experiments performed on the indicated cell fractions, representing a gradient of pre-BCR signaling. Shown are the relative interaction frequencies (average of two replicate experiments) for the iEκ viewpoint per 100 kb region across the *Ig*κ locus, plotted as line graphs. The bottom graph shows an overlay of the WT and *Btk* ^−/−^ *Slp65* ^−/−^ pre-B cell interaction frequencies. The ∼8.4 Mb region containing the *Ig*κ locus (yellow shading) and flanking regions (cyan shading) is depicted. Pre-B cell fractions were FACS-purified from the indicated mice on a VH81x transgenic *Rag1* ^−/−^ background (see Figure 1 for gating strategy).
(TIF)

Figure S4 **Locus-wide 3C-Seq analysis of the** ***Ig*** **κ region (3′Eκ viewpoint) plotted as line graphs.** Overview of long-range interactions revealed by 3C-Seq experiments performed on the indicated cell fractions, representing a gradient of pre-BCR signaling. Shown are the relative interaction frequencies (average of two replicate experiments) for the 3′Eκ viewpoint per 100 kb region across the *Ig*κ locus, plotted as line graphs. The bottom graph shows an overlay of the WT and *Btk* ^−/−^ *Slp65* ^−/−^ pre-B cell interaction frequencies. The ∼8.4 Mb region containing the *Ig*κ locus (yellow shading) and flanking regions (cyan shading) is depicted. Pre-B cell fractions were FACS-purified from the indicated mice on a VH81x transgenic *Rag1* ^−/−^ background (see Figure 1 for gating strategy).
(TIF)

Figure S5 **Selected zoom-in pictures of the 3C-seq data in the** ***Ig*** **κ locus and its upstream and downstream regions.** (A) Map of the ∼8.4 Mb genomic region containing the *Ig*κ locus (yellow shading) and flanking regions (cyan shading). Chromosomal coordinates and gene and viewpoint locations are also depicted. Locations of the zoom-in regions shown in (B) are represented as colored rectangles (red, upstream; green, downstream; dark grey, *Ig*κ locus). (B) Average interaction frequencies per BglII fragment (average of two replicate experiments) of the three regulatory elements with selected regions upstream, downstream, and within the *Ig*κ locus. Data from the five B cell precursor fractions representing a pre-BCR signaling gradient are shown. Complete locus-wide 3C-seq data can be found in Figure 3 (plotted per individual BglII fragment) or Figures S2, S3, and S4 (plotted per 100 kb region). enh., enhancers.
(TIF)

Figure S6 **Phenotypic characterization of cultured pre-pro-B, pro-B, and pre-B cells.** Representative FACS analysis of CD43/CD19 (A) and CD2/CD19 (B) surface marker expression on IL-7 cultured *E2a* ^−/−^ pre-pro-B, *Rag1* ^−/−^ pro-B, and VH81x *Rag1* ^−/−^ pre-B cells prior to 3D-FISH experiments.
(TIF)

Figure S7 **Pre-BCR signaling is associated with an increase in the ratio of interactions inside the** ***Ig*** **κ locus over interactions outside the** ***Ig*** **κ locus.** The differential effects of pre-BCR signaling on long-range chromatin interactions of the iEκ, 3′Eκ, and Sis elements, as measured in 3C-seq datasets, were quantified (see Figure 4A). For all three regulatory elements, the *y*-axis shows the ratio of the average interaction frequencies per BglII fragment inside the ∼3.2 Mb *Igκ* locus over the average interaction frequencies per BglII fragment in the flanking regions (∼2.0 Mb upstream together with 3.2 Mb downstream). Analyses of the five groups of B cell precursors, representing a gradient of pre-BCR signaling, are shown, revealing that pre-BCR signaling is associated with a preference for interaction with fragments inside the V_κ_ region over fragments outside the V_κ_ region.
(TIF)

Figure S8 **Interaction frequencies of κ regulatory elements with nonfunctional V_κ_ genes as measured in B cell fractions representing a pre-BCR signaling gradient.** Quantitative analysis of 3C-Seq datasets obtained for the five B cell precursor fractions representing a pre-BCR signaling gradient, using the three indicated κ regulatory elements as viewpoints. Average interaction frequencies within the V_κ_ region were determined for fragments that do not contain any V_κ_ gene (*“no V_κ_”*), those that contain a nonfunctional V_κ_ gene (*“pseudo V_κ_”*), and those that contain a functional V_κ_ gene (*“functional V_κ_”*). See Materials and Methods section for more details on analysis methods. Statistical significance was determined using a Mann–Whitney U test (n.s., not significant, *p*≥0.05).
(TIF)

Figure S9 **Correlations between the V_κ_ interaction profiles of the three κ regulatory elements.** Correlation plots of average interaction frequencies of the three regulatory elements with the 101 functional V_κ_ genes (A) or only the V_κ_3 gene family (B) are shown for WT pre-B cells (top, gray labels) versus *Btk* ^−/−^ *Slp65* ^−/−^ pre-B cells (bottom, yellow labels). Note that under conditions of low pre-BCR signaling (*Btk* ^−/−^ *Slp65* ^−/−^ pre-B cells) correlation strength is significantly reduced, with the exception of the correlation between de V_κ_3 interaction profiles of the Sis and iEκ elements.
(TIF)

Table S1 **Genes down-regulated in the absence of Btk and Slp65.**
(DOC)

Table S2 **Genes up-regulated in the absence of Btk and Slp65.**
(DOC)

Table S3 **Binding of E2a and Ikaros to the κ enhancers and V_κ_ genes in wild-type and Btk or Slp65-deficient pre-B cells.**
(DOC)

## Abbreviations

3′Eκ: 3′ κ enhancer
Btk: Bruton's tyrosine kinase
H3K4me2/3: histone H3 di- or trimethylated at lysine 4
iEκ: intronic κ enhancer
Ig: Immunoglobulin
pre-BCR: pre–B-cell receptor
Sis: silencer in intervening sequence
SLC: surrogate light chain
TF: transcription factor
V_κ_: Igκ variable region
