# Supplementary figures and images for: Pre-B Cell Receptor Signaling Induces Immunoglobulin κ Locus Accessibility by Functional Redistribution of Enhancer-Mediated Chromatin Interactions
Source: PLoS Biol. 2014 Feb 18;12(2):e1001791. doi: 10.1371/journal.pbio.1001791 (PMC3928034; doi:10.1371/journal.pbio.1001791)

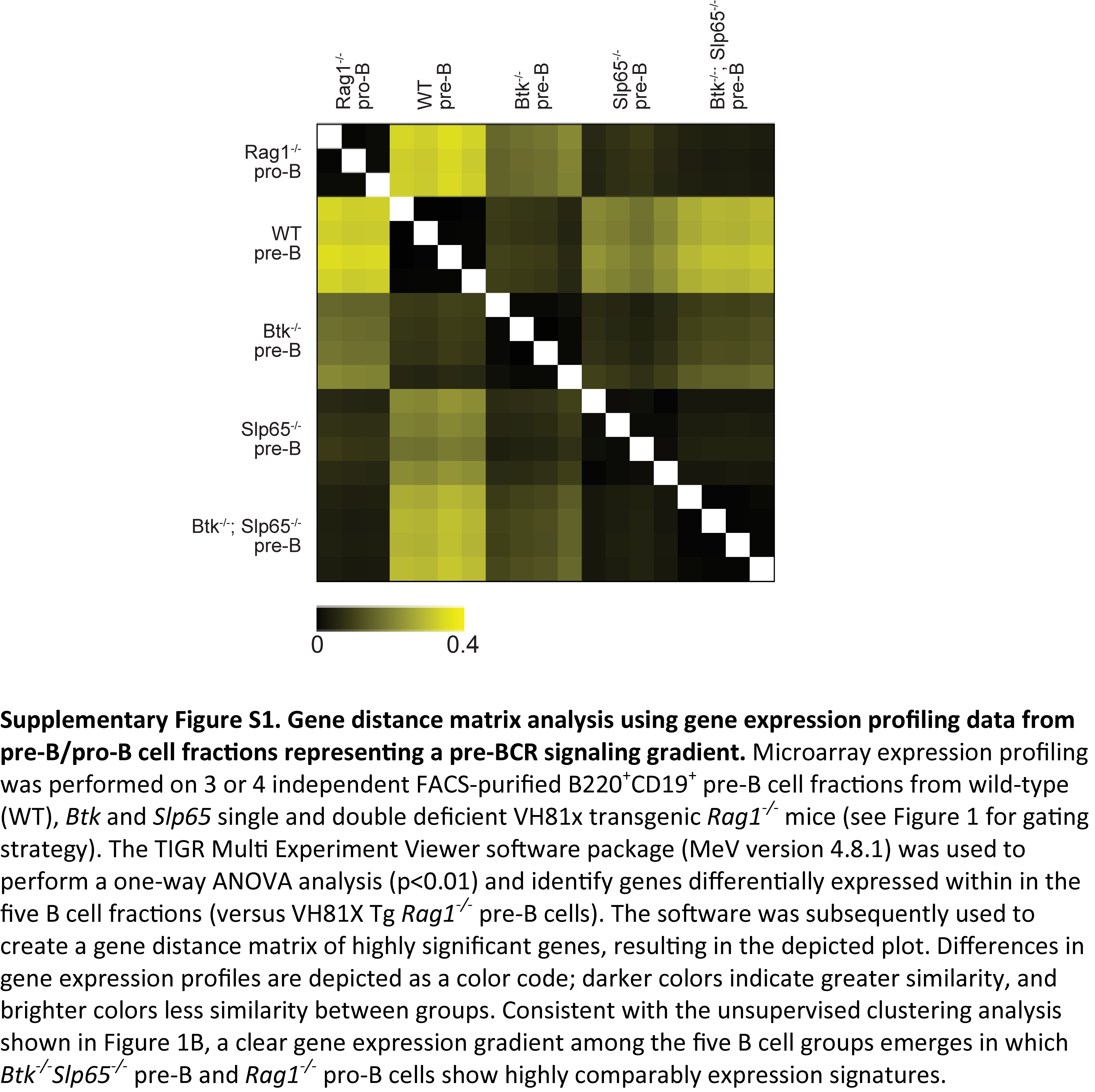

Supplement: Figure S1 — Gene distance matrix analysis using gene expression profiling data from pre-B/pro-B cell fractions representing a pre-BCR signaling gradient. Microarray expression profiling was performed on three or four independent FACS-purified B220+CD19+ pre-B cell fractions from wild-type (WT), Btk, and Slp65 single- and double-deficient VH81x transgenic Rag1 −/− mice (see Figure 1 for gating strategy). The TIGR Multi Experiment Viewer software package (MeV version 4.8.1) was used to perform a one-way ANOVA analysis (p<0.01) and identify genes differentially expressed within the five B-cell fractions (versus VH81X Tg Rag1 −/− pre-B cells). The software was subsequently used to create a gene distance matrix of highly significant genes, resulting in the depicted plot. Differences in gene expression profiles are depicted as a color code; darker colors indicate greater similarity, and brighter colors less similarity between groups. Consistent with the unsupervised clustering analysis shown in Figure 1B, a clear gene expression gradient among the five B cell groups emerges in which Btk −/− Slp65 −/− pre-B and Rag1 −/− pro-B cells show highly comparably expression signatures. (TIF) [file pbio.1001791.s001.tif]

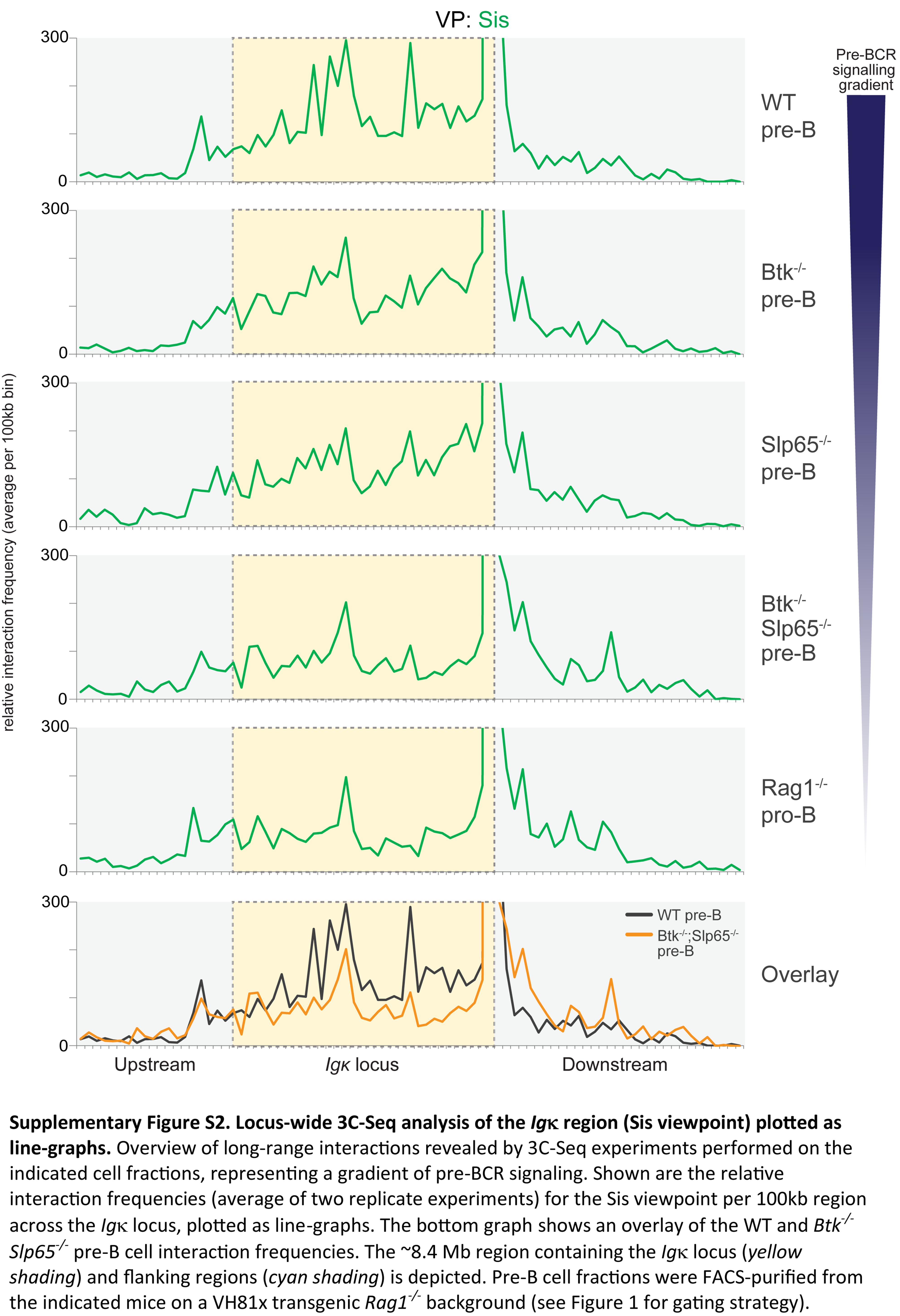

Supplement: Figure S2 — Locus-wide 3C-Seq analysis of the Ig κ region (Sis viewpoint) plotted as line graphs. Overview of long-range interactions revealed by 3C-Seq experiments performed on the indicated cell fractions, representing a gradient of pre-BCR signaling. Shown are the relative interaction frequencies (average of two replicate experiments) for the Sis viewpoint per 100 kb region across the Igκ locus, plotted as line graphs. The bottom graph shows an overlay of the WT and Btk −/− Slp65 −/− pre-B cell interaction frequencies. The ∼8.4 Mb region containing the Igκ locus (yellow shading) and flanking regions (cyan shading) is depicted. Pre-B cell fractions were FACS-purified from the indicated mice on a VH81x transgenic Rag1 −/− background (see Figure 1 for gating strategy). (TIF) [file pbio.1001791.s002.tif]

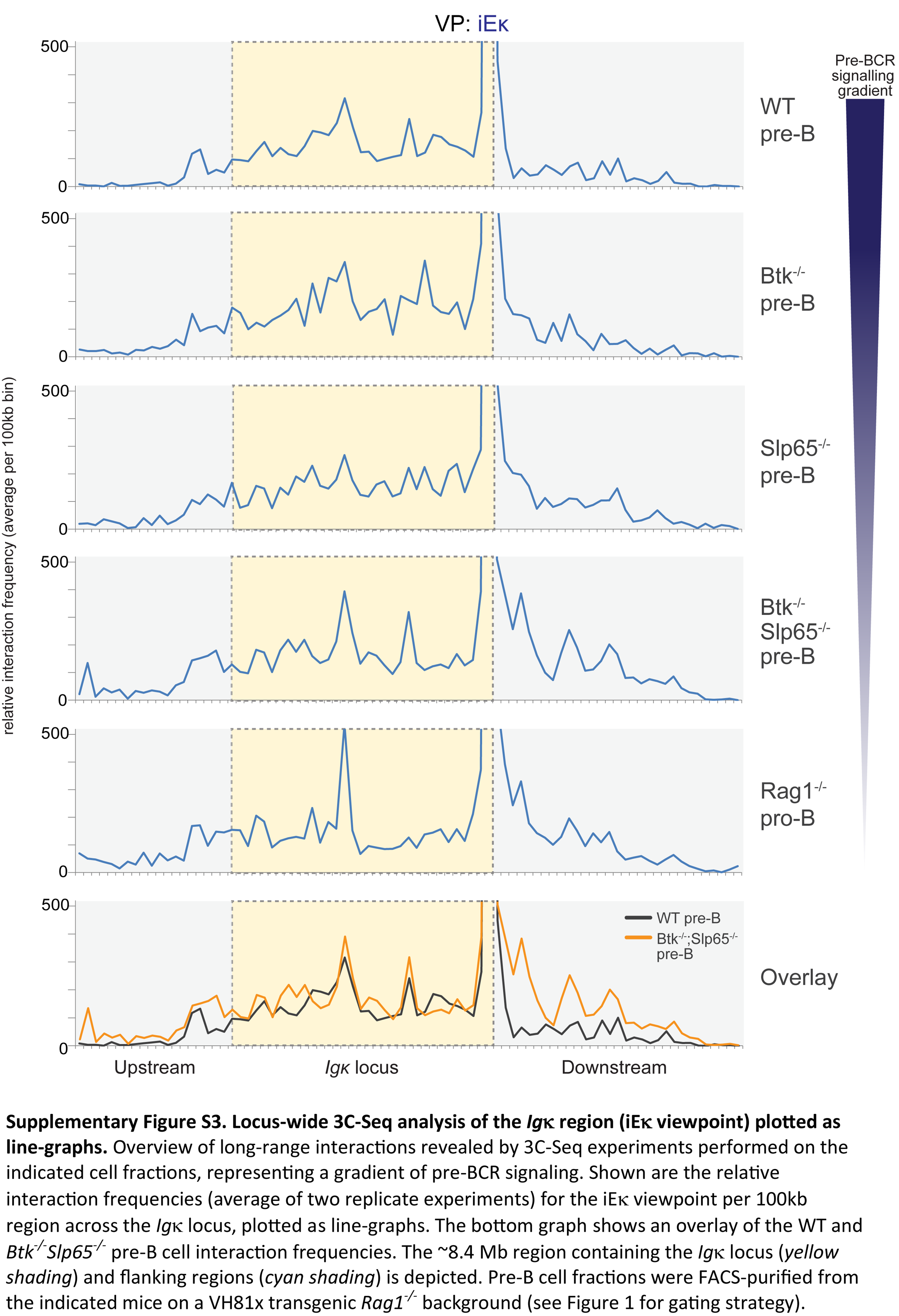

Supplement: Figure S3 — Locus-wide 3C-Seq analysis of the Ig κ region (iEκ viewpoint) plotted as line graphs. Overview of long-range interactions revealed by 3C-Seq experiments performed on the indicated cell fractions, representing a gradient of pre-BCR signaling. Shown are the relative interaction frequencies (average of two replicate experiments) for the iEκ viewpoint per 100 kb region across the Igκ locus, plotted as line graphs. The bottom graph shows an overlay of the WT and Btk −/− Slp65 −/− pre-B cell interaction frequencies. The ∼8.4 Mb region containing the Igκ locus (yellow shading) and flanking regions (cyan shading) is depicted. Pre-B cell fractions were FACS-purified from the indicated mice on a VH81x transgenic Rag1 −/− background (see Figure 1 for gating strategy). (TIF) [file pbio.1001791.s003.tif]

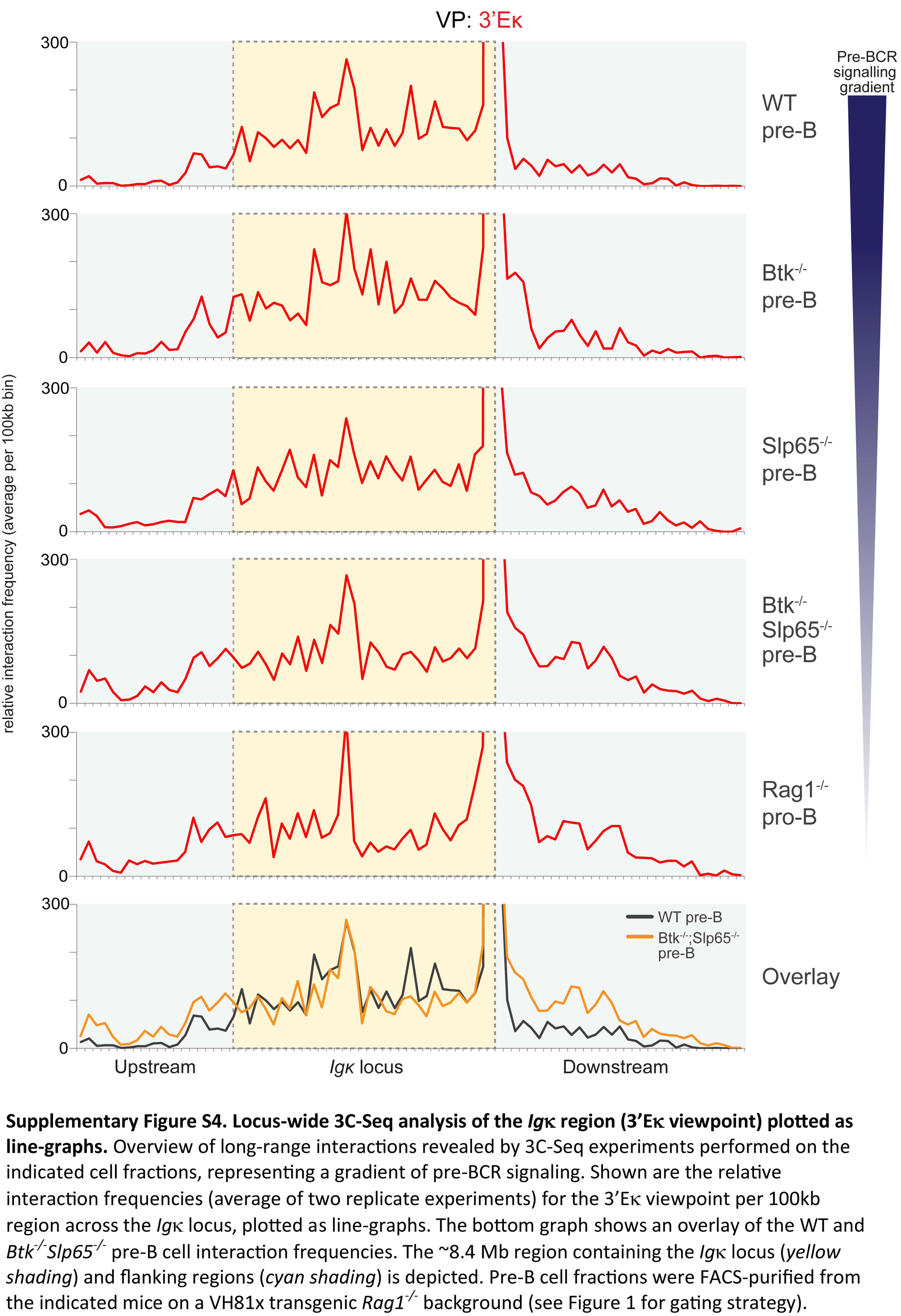

Supplement: Figure S4 — Locus-wide 3C-Seq analysis of the Ig κ region (3′Eκ viewpoint) plotted as line graphs. Overview of long-range interactions revealed by 3C-Seq experiments performed on the indicated cell fractions, representing a gradient of pre-BCR signaling. Shown are the relative interaction frequencies (average of two replicate experiments) for the 3′Eκ viewpoint per 100 kb region across the Igκ locus, plotted as line graphs. The bottom graph shows an overlay of the WT and Btk −/− Slp65 −/− pre-B cell interaction frequencies. The ∼8.4 Mb region containing the Igκ locus (yellow shading) and flanking regions (cyan shading) is depicted. Pre-B cell fractions were FACS-purified from the indicated mice on a VH81x transgenic Rag1 −/− background (see Figure 1 for gating strategy). (TIF) [file pbio.1001791.s004.tif]

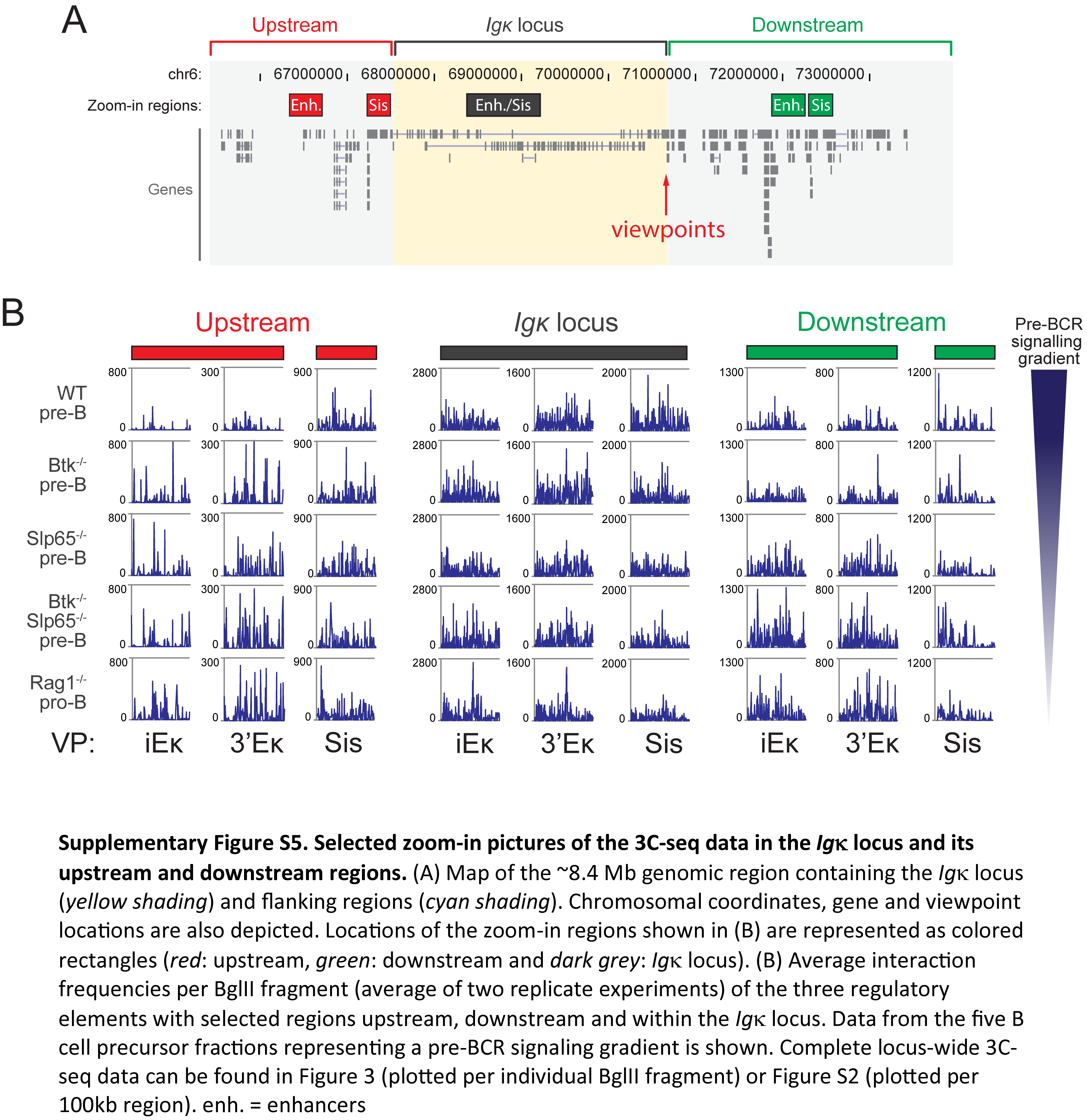

Supplement: Figure S5 — Selected zoom-in pictures of the 3C-seq data in the Ig κ locus and its upstream and downstream regions. (A) Map of the ∼8.4 Mb genomic region containing the Igκ locus (yellow shading) and flanking regions (cyan shading). Chromosomal coordinates and gene and viewpoint locations are also depicted. Locations of the zoom-in regions shown in (B) are represented as colored rectangles (red, upstream; green, downstream; dark grey, Igκ locus). (B) Average interaction frequencies per BglII fragment (average of two replicate experiments) of the three regulatory elements with selected regions upstream, downstream, and within the Igκ locus. Data from the five B cell precursor fractions representing a pre-BCR signaling gradient are shown. Complete locus-wide 3C-seq data can be found in Figure 3 (plotted per individual BglII fragment) or Figures S2, S3, and S4 (plotted per 100 kb region). enh., enhancers. (TIF) [file pbio.1001791.s005.tif]

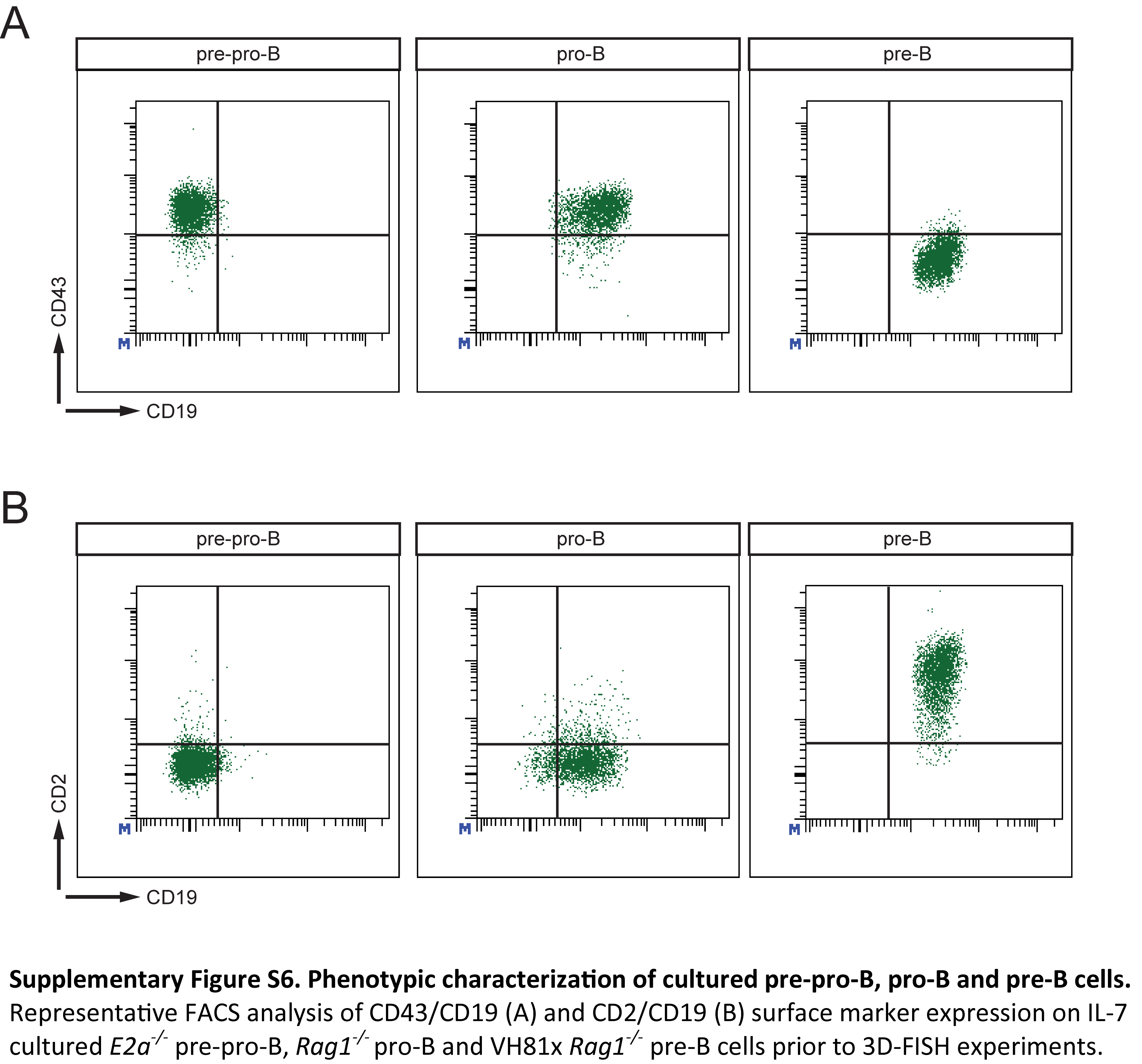

Supplement: Figure S6 — Phenotypic characterization of cultured pre-pro-B, pro-B, and pre-B cells. Representative FACS analysis of CD43/CD19 (A) and CD2/CD19 (B) surface marker expression on IL-7 cultured E2a −/− pre-pro-B, Rag1 −/− pro-B, and VH81x Rag1 −/− pre-B cells prior to 3D-FISH experiments. (TIF) [file pbio.1001791.s006.tif]

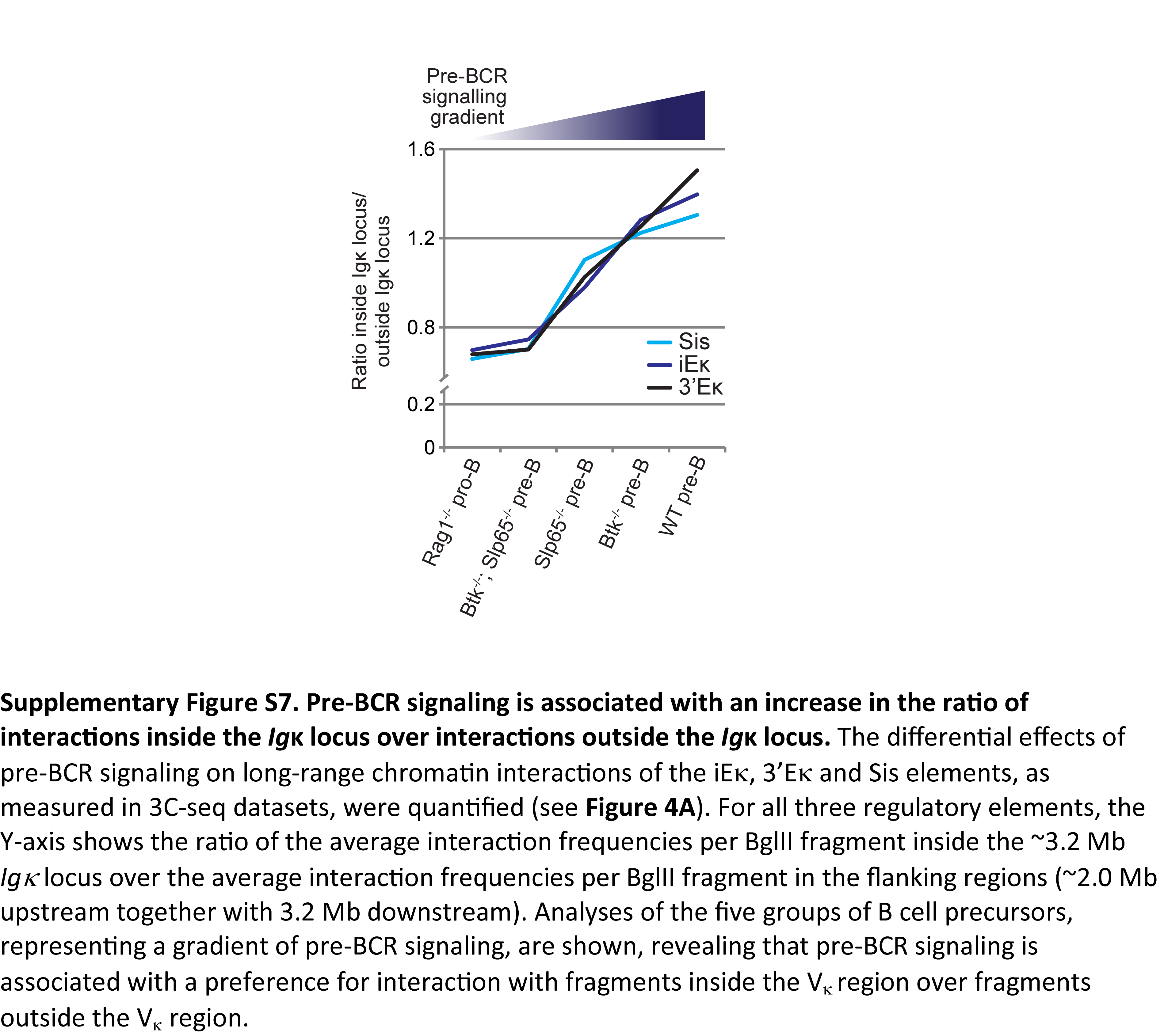

Supplement: Figure S7 — Pre-BCR signaling is associated with an increase in the ratio of interactions inside the Ig κ locus over interactions outside the Ig κ locus. The differential effects of pre-BCR signaling on long-range chromatin interactions of the iEκ, 3′Eκ, and Sis elements, as measured in 3C-seq datasets, were quantified (see Figure 4A). For all three regulatory elements, the y-axis shows the ratio of the average interaction frequencies per BglII fragment inside the ∼3.2 Mb Igκ locus over the average interaction frequencies per BglII fragment in the flanking regions (∼2.0 Mb upstream together with 3.2 Mb downstream). Analyses of the five groups of B cell precursors, representing a gradient of pre-BCR signaling, are shown, revealing that pre-BCR signaling is associated with a preference for interaction with fragments inside the Vκ region over fragments outside the Vκ region. (TIF) [file pbio.1001791.s007.tif]

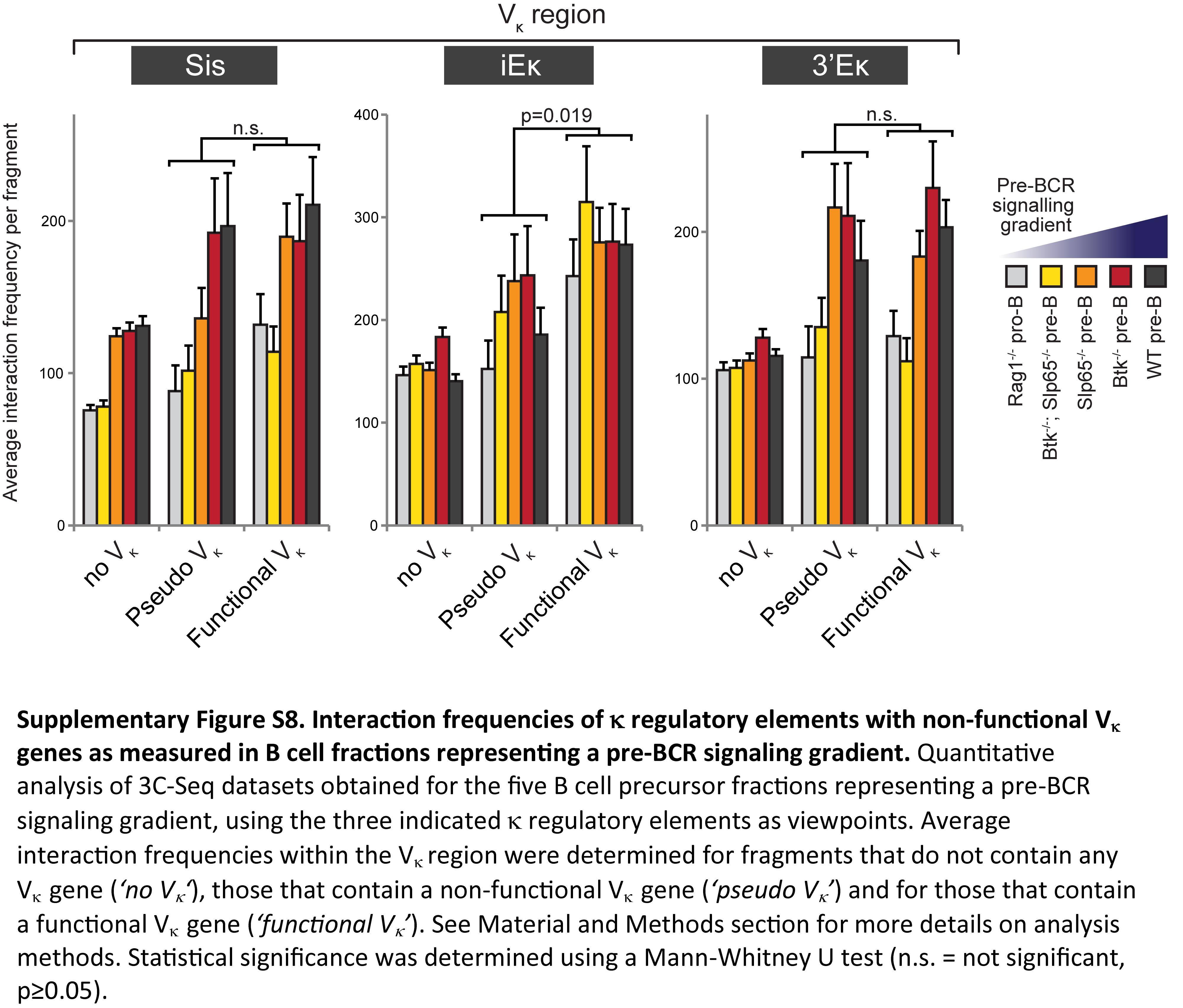

Supplement: Figure S8 — Interaction frequencies of κ regulatory elements with nonfunctional Vκ genes as measured in B cell fractions representing a pre-BCR signaling gradient. Quantitative analysis of 3C-Seq datasets obtained for the five B cell precursor fractions representing a pre-BCR signaling gradient, using the three indicated κ regulatory elements as viewpoints. Average interaction frequencies within the Vκ region were determined for fragments that do not contain any Vκ gene (“no Vκ”), those that contain a nonfunctional Vκ gene (“pseudo Vκ”), and those that contain a functional Vκ gene (“functional Vκ”). See Materials and Methods section for more details on analysis methods. Statistical significance was determined using a Mann–Whitney U test (n.s., not significant, p≥0.05). (TIF) [file pbio.1001791.s008.tif]

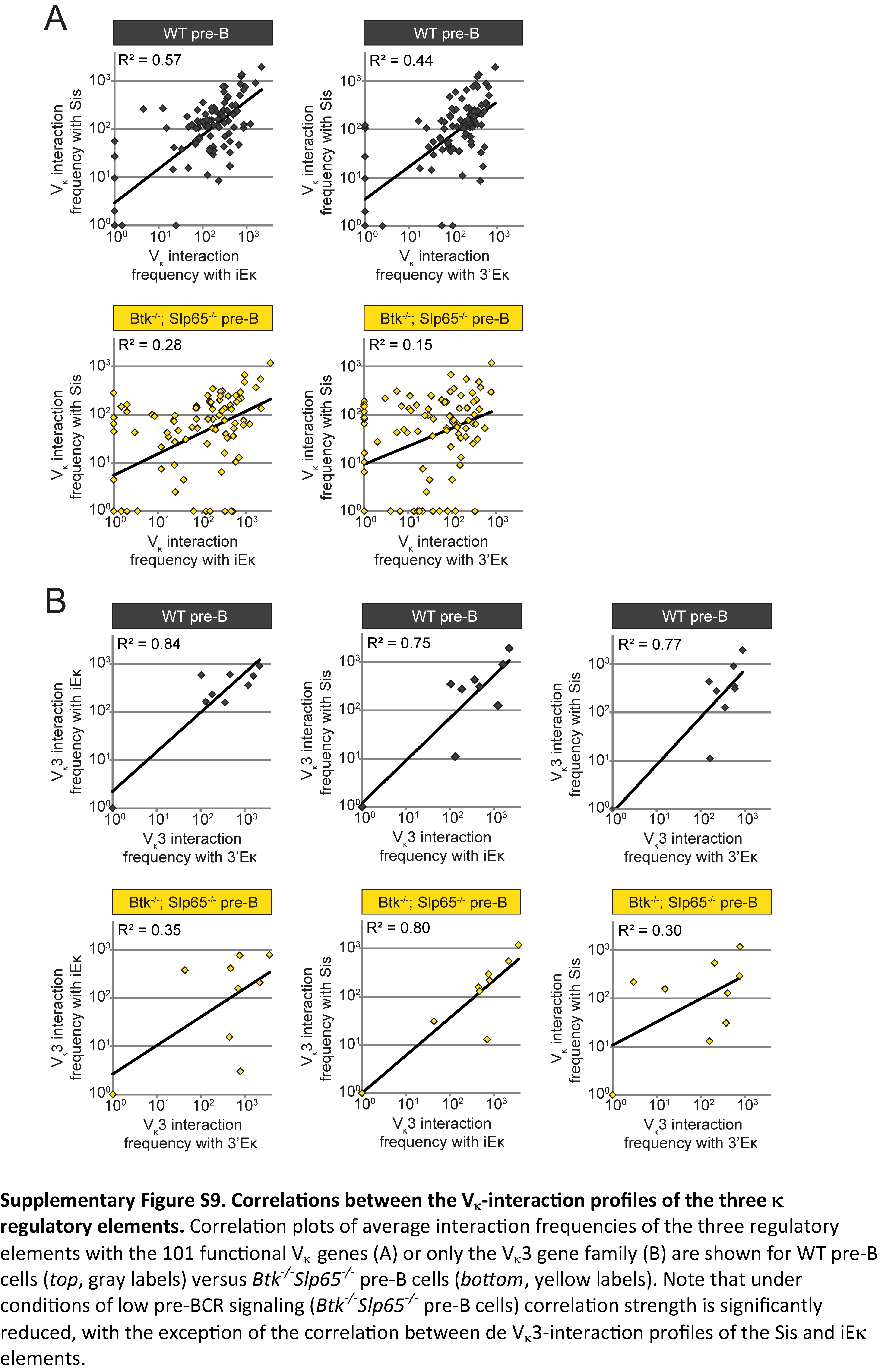

Supplement: Figure S9 — Correlations between the Vκ interaction profiles of the three κ regulatory elements. Correlation plots of average interaction frequencies of the three regulatory elements with the 101 functional Vκ genes (A) or only the Vκ3 gene family (B) are shown for WT pre-B cells (top, gray labels) versus Btk −/− Slp65 −/− pre-B cells (bottom, yellow labels). Note that under conditions of low pre-BCR signaling (Btk −/− Slp65 −/− pre-B cells) correlation strength is significantly reduced, with the exception of the correlation between de Vκ3 interaction profiles of the Sis and iEκ elements. (TIF) [file pbio.1001791.s009.tif]
