# Supplementary material for: Pre-B Cell Receptor Signaling Induces Immunoglobulin κ Locus Accessibility by Functional Redistribution of Enhancer-Mediated Chromatin Interactions
Source: PLoS Biol. 2014 Feb 18;12(2):e1001791. doi: 10.1371/journal.pbio.1001791 (PMC3928034; doi:10.1371/journal.pbio.1001791)
Supplement: Table S1 — Genes down-regulated in the absence of Btk and Slp65. (DOC) [file pbio.1001791.s010.doc]

**Supplementary Table S1. Genes up regulated in the absence of Btk and Slp65.**

ANOVA analysis of genes differentially expressed between *Rag1^-/-^*pro-B cells and V_H_81X Tg *Rag1^-/-^*pre-B cells that were either wild-type (WT), *Btk^-/-^, Slp65^-/-^* or *Btk^-/-^Slp65^-/-^.*

| **ID**  **Probe set** | **Accession number** | **Gene** | **p-value** | **Fold Change**  **(Btk KO)** | **Fold Change**  **(Slp65 KO)** | **Fold Change (Btk/Slp65 KO)** | **Fold Change**  **(Rag1 KO)^a^** |
| --- | --- | --- | --- | --- | --- | --- | --- |
| **10538706** | BC137623 | Mmrn1 | 2.31E-06 | 1.48 | 20.71 | 33.44 | 30.04 |
| **10463123** | NM_009345 | Dntt | 4.26E-08 | 8.46 | 16.99 | 22.49 | 39.19 |
| **10590433** | BC048726 | 700048O20Rik | 2.12E-05 | 2.38 | 8.78 | 12.28 | 15.60 |
| **10597323** | NM_033264 | Arpp21 | 2.44E-08 | 3.41 | 7.62 | 10.10 | 10.92 |
| **10357833** | NM_213616 | Atp2b4 | 6.11E-05 | 1.89 | 4.97 | 9.74 | 7.54 |
| **10538126** | NM_174990 | Gimap4 | 2.29E-03 | 1.20 | 13.36 | 7.94 | 17.76 |
| **10346168** | NM_011487 | Stat4 | 1.78E-04 | 2.32 | 6.21 | 7.65 | 9.66 |
| **10491091** | NM_009425 | Tnfsf10 | 1.04E-04 | 4.92 | 7.25 | 7.21 | 9.03 |
| **10466606** | NM_010730 | Anxa1 | 9.15E-05 | 2.94 | 5.31 | 6.52 | 5.80 |
| **10550877** | NM_008433 | Kcnn4 | 2.53E-05 | 2.20 | 5.57 | 6.44 | 6.85 |
| **10438064** | NM_016982 | Vpreb1 | 7.58E-06 | 5.52 | 6.80 | 6.32 | 5.73 |
| **10396170** | NM_028127 | Frmd6 | 4.61E-06 | 1.71 | 3.48 | 6.17 | 5.42 |
| **10396402** | NM_008856 | Prkch | 5.55E-06 | 3.33 | 4.95 | 6.15 | 5.79 |
| **10471486** | NM_001146350 | Eng | 2.17E-07 | 3.22 | 6.14 | 6.14 | 5.48 |
| **10491952** | NM_174995 | Mgst2 | 1.27E-04 | 1.84 | 6.86 | 5.90 | 7.37 |
| **10360053** | NM_025557 | Pcp4l1 | 1.52E-05 | 3.71 | 8.87 | 5.62 | 7.02 |
| **10506488** | NM_080555 | Ppap2b | 1.46E-06 | 1.38 | 6.02 | 5.47 | 4.82 |
| **10443786** | NM_008804 | Pde9a | 1.99E-05 | 1.53 | 4.15 | 5.42 | 4.73 |
| **10432675** | ENSMUST00000089252 | I730030J21Rik | 8.42E-05 | 1.96 | 7.18 | 5.15 | 4.24 |
| **10567825** | NM_010689 | Lat | 8.42E-04 | 3.64 | 3.69 | 5.11 | 2.09 |
| **10539933** | NM_153162 | Txnrd3 | 5.69E-03 | 2.67 | 3.22 | 5.06 | 2.93 |
| **10586017** | NM_028283 | Uaca | 3.22E-05 | 3.03 | 4.80 | 4.96 | 4.42 |
| **10592888** | NM_007551 | Cxcr5 | 6.88E-06 | 1.16 | 6.68 | 4.92 | 8.81 |
| **10542355** | NM_010128 | Emp1 | 7.68E-05 | 1.67 | 2.74 | 4.82 | 8.57 |
| **10437272** | NM_001081280 | Nlrc3 | 5.41E-05 | 2.39 | 3.64 | 4.81 | 3.12 |
| **10468898** | NM_001159649 | Lax1 | 1.33E-04 | 1.68 | 4.18 | 4.65 | 4.91 |
| **10595798** | BC035950 | BC043934 | 2.29E-04 | 1.08 | 4.82 | 4.55 | 7.32 |
| **10472846** | NM_172665 | Pdk1 | 7.75E-04 | 2.13 | 3.08 | 4.47 | 4.85 |
| **10380927** | NM_010346 | Grb7 | 1.17E-05 | 2.43 | 14.08 | 4.40 | 7.86 |
| **10505517** | NM_021297 | Tlr4 | 8.57E-05 | 2.04 | 6.09 | 4.39 | 4.57 |
| **10367734** | NM_177387 | Ust | 6.32E-05 | 1.40 | 4.28 | 4.31 | 6.80 |
| **10383756** | NM_030694 | Ifitm2 | 2.34E-03 | 2.40 | 3.94 | 4.31 | 3.99 |
| **10354019** | BC072639 | 2010300C02Rik | 3.60E-05 | 1.20 | 3.81 | 4.27 | 6.84 |
| **10553299** | NM_030694 | Ifitm2 | 4.05E-04 | 2.45 | 3.66 | 4.27 | 3.68 |
| **10574259** | NM_018882 | Gpr56 | 3.10E-03 | 2.60 | 3.54 | 4.20 | 3.15 |
| **10438060** | ENSMUST00000100136 | Igll1 | 2.17E-04 | 3.38 | 3.81 | 4.17 | 3.69 |
| **10383731** | NM_001159284 | Smtn | 1.45E-06 | 1.62 | 2.75 | 4.08 | 1.99 |
| **10543572** | NM_011829 | Impdh1 | 8.73E-05 | 2.48 | 3.77 | 4.05 | 3.06 |
| **10574288** | NM_001042715 | Ccdc135 | 5.18E-04 | 2.55 | 3.32 | 3.97 | 3.39 |
| **10527638** | NM_009663 | Alox5ap | 1.20E-03 | 2.64 | 5.11 | 3.85 | 3.44 |
| **10464642** | BC023699 | Atpgd1 | 3.45E-03 | 1.95 | 2.63 | 3.77 | 1.81 |
| **10345509** | NM_009539 | Zap70 | 3.41E-04 | 3.51 | 4.00 | 3.77 | 1.70 |
| **10496569** | NM_145545 | Gbp6 | 4.58E-04 | 2.12 | 4.87 | 3.76 | 4.74 |
| **10410547** | NM_028186 | Nkd2 | 8.23E-04 | 1.24 | 3.41 | 3.73 | 2.27 |
| **10463632** | NM_029186 | Tmem180 | 6.98E-03 | 1.50 | 3.06 | 3.73 | 2.73 |
| **10440738** | NM_009384 | Tiam1 | 7.92E-06 | 1.78 | 3.46 | 3.73 | 2.42 |
| **10374455** | NM_033523 | Spred2 | 1.47E-05 | 1.85 | 3.88 | 3.70 | 2.72 |
| **10416340** | NM_008115 | Gfra2 | 4.70E-06 | 2.93 | 3.22 | 3.48 | 2.41 |
| **10524229** | ENSMUST00000059200 | A630023P12Rik | 8.80E-04 | 1.12 | 3.58 | 3.44 | 8.66 |
| **10497149** | NM_026582 | Gpr177 | 3.43E-03 | 2.18 | 3.94 | 3.44 | 3.49 |
| **10388545** | NM_198895 | Abr | 6.54E-09 | 2.03 | 2.92 | 3.38 | 2.91 |
| **10399148** | NM_175930 | Rapgef5 | 1.62E-04 | 2.52 | 3.06 | 3.36 | 2.62 |
| **10377286** | NM_001081566 | Pik3r6 | 7.23E-06 | 1.41 | 2.58 | 3.35 | 2.64 |
| **10460263** | NM_027857 | Acy3 | 3.24E-07 | 1.82 | 3.46 | 3.28 | 2.33 |
| **10588495** | NM_153459 | Dusp7 | 2.59E-03 | 1.68 | 3.36 | 3.26 | 1.97 |
| **10554574** | NM_145375 | Tm6sf1 | 1.35E-03 | 1.96 | 3.04 | 3.25 | 3.02 |
| **10388047** | NM_001004142 | Nlrp1a | 6.67E-03 | 1.41 | 2.60 | 3.18 | 3.08 |
| **10464647** | NM_178650 | Tbc1d10c | 6.52E-04 | 1.59 | 2.73 | 3.14 | 2.23 |
| **10477237** | BC020535 | BC020535 | 3.55E-05 | -1.14 | 2.04 | 3.13 | 2.88 |
| **10557591** | NM_008400 | Itgal | 5.15E-03 | 1.81 | 3.12 | 3.11 | 2.00 |
| **10469255** | NM_008859 | Prkcq | 1.36E-04 | 1.34 | 3.43 | 3.10 | 3.18 |
| **10596190** | NM_001002896 | Bfsp2 | 4.90E-03 | 2.51 | 3.50 | 3.10 | 1.66 |
| **10406031** | NM_145376 | Lpcat1 | 6.99E-05 | 1.34 | 2.45 | 3.06 | 2.12 |
| **10481349** | NM_133501 | Ntng2 | 1.07E-05 | 1.78 | 3.07 | 3.06 | 1.36 |
| **10493421** | BC088990 | Fam189b | 4.30E-04 | 1.60 | 2.71 | 3.03 | 2.00 |
| **10494551** | NM_019800 | Acp6 | 1.12E-03 | 2.26 | 2.91 | 3.03 | 2.68 |
| **10375145** | NM_010696 | Lcp2 | 1.03E-04 | 2.68 | 4.90 | 3.02 | 2.51 |
| **10365098** | NM_009325 | Tbxa2r | 2.98E-04 | 1.38 | 3.12 | 3.02 | 1.80 |
| **10512480** | NM_019436 | Sit1 | 2.34E-04 | 2.24 | 4.04 | 3.02 | 2.86 |
| **10441565** | NM_011299 | Rps6ka2 | 6.49E-03 | 1.10 | 2.03 | 2.98 | 2.31 |
| **10418702** | NM_011894 | Sh3bp5 | 2.19E-06 | 1.70 | 2.50 | 2.92 | 2.21 |
| **10373027** | NM_025982 | Tspan31 | 1.07E-04 | 1.95 | 3.45 | 2.84 | 2.77 |
| **10507040** | NM_026470 | Spata6 | 1.97E-03 | 1.33 | 2.56 | 2.83 | 3.57 |
| **10404885** | NM_025508 | Gmpr | 7.47E-03 | 1.33 | 2.68 | 2.78 | 2.79 |
| **10590438** | BC113767 | C730027P07Rik | 9.12E-03 | 1.33 | 2.11 | 2.73 | 2.44 |
| **10502081** | NM_007934 | Enpep | 4.57E-03 | 7.83 | 6.36 | 2.73 | 3.05 |
| **10492815** | NM_177260 | Tmem154 | 1.28E-05 | 1.59 | 3.94 | 2.71 | 2.41 |
| **10362596** | NM_001122893 | Fyn | 5.08E-04 | 1.66 | 2.52 | 2.69 | 1.69 |
| **10445746** | NM_021406 | Trem1 | 4.35E-03 | 1.14 | 1.59 | 2.66 | 2.71 |
| **10550972** | NM_001102613 | Phldb3 | 1.28E-03 | 1.46 | 2.77 | 2.64 | 1.63 |
| **10406111** | NM_011390 | Slc12a7 | 2.26E-03 | 1.17 | 2.55 | 2.64 | 2.45 |
| **10498852** | NM_212452 | Rxfp1 | 1.02E-03 | 1.39 | 2.82 | 2.63 | 2.22 |
| **10496091** | NM_010703 | Lef1 | 2.63E-05 | 1.97 | 2.49 | 2.62 | 2.26 |
| **10526459** | NM_133914 | Rasa4 | 4.19E-04 | 2.48 | 2.71 | 2.62 | 1.29 |
| **10371220** | NM_010304 | Gna15 | 7.76E-03 | 2.02 | 2.72 | 2.56 | 2.23 |
| **10604175** | NM_172930 | Fam70a | 2.35E-03 | 1.50 | 3.09 | 2.55 | 2.82 |
| **10456492** | BC096371 | D18Ertd653e | 5.75E-05 | 1.06 | 1.90 | 2.55 | 3.00 |
| **10445241** | NM_178589 | Tnfrsf21 | 3.89E-04 | 1.91 | 2.30 | 2.53 | 1.68 |
| **10553598** | NM_011370 | Cyfip1 | 2.45E-04 | 1.73 | 2.90 | 2.53 | 3.01 |
| **10594645** | NM_173413 | Rab8b | 5.55E-04 | 1.63 | 2.37 | 2.52 | 2.53 |
| **10571715** | NM_177304 | Enpp6 | 5.30E-03 | 1.88 | 4.16 | 2.49 | 1.84 |
| **10472097** | NM_172409 | Fmnl2 | 4.33E-03 | 1.17 | 2.45 | 2.49 | 3.11 |
| **10388185** | NM_177776 | Smtnl2 | 1.27E-04 | 1.24 | 3.60 | 2.48 | 3.23 |
| **10388430** | NM_011340 | Serpinf1 | 2.78E-05 | 2.05 | 2.78 | 2.48 | 1.82 |
| **10427997** | NM_027496 | Ankrd33b | 2.59E-03 | 1.37 | 2.90 | 2.46 | 3.29 |
| **10385747** | NM_199299 | Phf15 | 2.21E-03 | 1.59 | 1.99 | 2.46 | 2.21 |
| **10495147** | NM_001093754 | Dennd2d | 2.10E-03 | 1.24 | 2.37 | 2.46 | 1.99 |
| **10465132** | NM_011379 | Sipa1 | 5.02E-04 | 1.47 | 2.01 | 2.45 | 1.28 |
| **10567043** | NM_025846 | Rras2 | 4.11E-03 | 1.93 | 2.40 | 2.44 | 3.27 |
| **10348354** | NM_201644 | Ugt1a9 | 2.49E-03 | 1.20 | 1.40 | 2.44 | 2.12 |
| **10425092** | NM_028195 | Cyth4 | 5.58E-04 | 1.73 | 2.26 | 2.40 | 1.77 |
| **10358565** | NM_001024720 | Hmcn1 | 2.11E-04 | 1.26 | 3.46 | 2.40 | 2.27 |
| **10388749** | NM_009423 | Traf4 | 1.37E-03 | 1.58 | 2.02 | 2.39 | 1.64 |
| **10550956** | NM_023154 | Ethe1 | 3.76E-03 | 1.22 | 2.87 | 2.37 | 2.93 |
| **10538640** | NM_011920 | Abcg2 | 9.19E-03 | 1.55 | 2.85 | 2.36 | 2.32 |
| **10407327** | NM_010330 | Emb | 8.70E-03 | 1.13 | 4.13 | 2.35 | 5.79 |
| **10400844** | NM_133198 | Pygl | 9.85E-03 | 1.57 | 3.59 | 2.34 | 2.36 |
| **10443244** | NM_181413 | Anks1 | 1.37E-04 | 1.42 | 1.78 | 2.34 | 1.69 |
| **10350102** | NM_177081 | Ptpn7 | 2.10E-04 | 1.38 | 2.23 | 2.34 | 2.40 |
| **10502748** | NM_001081298 | Lphn2 | 1.43E-03 | -1.23 | 1.74 | 2.31 | 2.35 |
| **10374727** | NM_016707 | Bcl11a | 1.03E-05 | 2.04 | 2.05 | 2.31 | 1.23 |
| **10480145** | NM_009105 | Rsu1 | 7.43E-04 | 1.45 | 2.26 | 2.30 | 2.35 |
| **10542340** | BC137674 | 8430419L09Rik | 4.01E-04 | 1.35 | 2.04 | 2.29 | 1.72 |
| **10559248** | NM_020286 | Tspan32 | 5.42E-03 | 1.23 | 2.34 | 2.22 | 1.99 |
| **10397763** | BC066161 | 9030617O03Rik | 2.62E-03 | 1.26 | 2.18 | 2.20 | 1.81 |
| **10435288** | NM_010739 | Muc13 | 6.04E-04 | 1.07 | 1.58 | 2.19 | 2.53 |
| **10479463** | NM_183161 | Slc17a9 | 1.27E-04 | 2.15 | 2.52 | 2.19 | 1.39 |
| **10537834** | NM_133674 | Arhgef5 | 9.58E-03 | 1.22 | 1.78 | 2.19 | 2.34 |
| **10379765** | NM_009330 | Hnf1b | 7.15E-03 | 1.34 | 1.79 | 2.18 | 1.60 |
| **10584762** | NM_030256 | Bcl9l | 8.57E-04 | 1.33 | 1.73 | 2.18 | 1.21 |
| **10412882** | NM_009380 | Thrb | 1.91E-04 | 1.36 | 2.03 | 2.16 | 3.16 |
| **10367600** | NM_007956 | Esr1 | 5.42E-03 | 1.12 | 1.85 | 2.13 | 1.85 |
| **10369993** | NM_133994 | Gstt3 | 1.53E-03 | 1.90 | 2.87 | 2.10 | 1.66 |
| **10409567** | NM_145976 | Tifab | 2.76E-05 | 2.06 | 2.42 | 2.10 | 1.14 |
| **10584941** | NM_011792 | Bace1 | 4.76E-03 | 1.83 | 2.73 | 2.09 | 1.86 |
| **10428004** | NM_026153 | Ankrd33b | 9.26E-03 | 1.23 | 2.33 | 2.09 | 2.25 |
| **10467041** | NM_018830 | Asah2 | 4.89E-03 | 1.27 | 2.09 | 2.07 | 2.47 |
| **10377612** | NM_023564 | Plscr3 | 8.38E-04 | 1.45 | 2.07 | 2.06 | 1.71 |
| **10423825** | NM_008056 | Fzd6 | 1.74E-03 | 1.24 | 2.26 | 2.03 | 3.32 |
| **10443749** | NM_177823 | Ubash3a | 3.69E-04 | 2.49 | 2.73 | 2.00 | 1.38 |
| **10567626** | NM_028758 | Gga2 | 5.28E-03 | 1.21 | 1.96 | 2.00 | 1.15 |
| **10561073** | NM_177102 | Tmem91 | 5.17E-04 | 1.21 | 1.39 | 1.98 | 2.71 |
| **10485624** | NM_178695 | Prrg4 | 6.39E-03 | 1.31 | 2.53 | 1.98 | 2.95 |
| **10372781** | NM_028679 | Irak3 | 6.12E-03 | 1.35 | 1.92 | 1.95 | 1.80 |
| **10580061** | NM_016671 | Il27ra | 8.54E-04 | 1.39 | 1.85 | 1.94 | 1.53 |
| **10407097** | NM_011056 | Pde4d | 1.60E-04 | 1.38 | 1.77 | 1.93 | 1.76 |
| **10584883** | NM_022004 | Fxyd6 | 4.93E-03 | 1.16 | 1.99 | 1.93 | 3.14 |
| **10592655** | NM_027144 | Arhgef12 | 2.29E-04 | 1.33 | 2.34 | 1.92 | 1.27 |
| **10468992** | NM_172475 | Frmd4a | 2.49E-03 | 1.37 | 1.80 | 1.91 | 1.62 |
| **10451198** | NM_001025250 | Vegfa | 1.40E-04 | 2.11 | 2.01 | 1.86 | 1.08 |
| **10532944** | NM_175403 | Mlec | 2.36E-03 | 1.48 | 2.03 | 1.85 | 1.78 |
| **10485466** | NM_009804 | Cat | 4.29E-03 | 1.53 | 2.05 | 1.85 | 2.28 |
| **10358670** | NM_001024720 | Hmcn1 | 6.15E-03 | 1.09 | 2.36 | 1.84 | 2.39 |
| **10464612** | NM_144532 | Cabp4 | 6.17E-04 | 1.18 | 1.98 | 1.84 | 2.04 |
| **10357604** | NM_019777 | Ikbke | 5.33E-03 | 1.35 | 2.04 | 1.81 | 1.99 |
| **10600921** | NM_199018 | Stard8 | 2.34E-04 | 1.13 | 2.22 | 1.80 | 2.17 |
| **10345241** | NM_134448 | Dst | 1.08E-04 | 1.08 | 2.37 | 1.78 | 2.47 |
| **10452793** | NM_027864 | Galnt14 | 1.21E-03 | 1.94 | 1.67 | 1.76 | 1.21 |
| **10458278** | BC064044 | 010001M09Rik | 8.86E-03 | 1.25 | 2.25 | 1.75 | 3.24 |
| **10534253** | NM_001081462 | Gtf2ird1 | 8.63E-03 | 1.20 | 2.08 | 1.74 | 1.95 |
| **10454580** | NM_009668 | Bin1 | 4.21E-03 | 1.29 | 1.65 | 1.72 | 1.61 |
| **10555027** | NM_010248 | Gab2 | 8.06E-04 | 1.19 | 1.97 | 1.72 | 1.89 |
| **10493076** | NM_021309 | Sh2d2a | 1.00E-03 | 1.06 | 1.57 | 1.71 | 1.08 |
| **10370471** | NM_053014 | Agpat3 | 6.22E-03 | 1.30 | 1.82 | 1.71 | 1.39 |
| **10365518** | NM_175331 | Nt5dc3 | 1.07E-03 | -1.08 | 1.55 | 1.60 | 1.91 |
| **10350630** | NM_022018 | Fam129a | 4.06E-03 | -1.17 | 2.07 | 1.54 | 2.10 |
| **10370072** | NM_133182 | Prmt2 | 8.21E-03 | 1.19 | 1.62 | 1.52 | 1.45 |
| **10502890** | NM_011372 | St6galnac3 | 8.41E-04 | 1.06 | 1.36 | 1.47 | 1.84 |
| **10435009** | NM_001033539 | Bex6 | 1.48E-03 | 1.29 | 2.99 | 1.47 | 1.98 |
| **10554863** | NM_001040085 | Sytl2 | 2.03E-05 | 1.01 | 1.15 | 1.47 | 2.85 |
| **10560709** | NM_027514 | Pvr | 4.78E-03 | 1.25 | 1.71 | 1.44 | 1.60 |
| **10421258** | NM_145978 | Pdlim2 | 4.05E-03 | 1.41 | 1.52 | 1.41 | 1.18 |
| **10469457** | NM_026162 | Plxdc2 | 2.04E-04 | 1.12 | 1.90 | 1.38 | 2.37 |
| **10439483** | NM_020260 | Cdgap | 7.96E-04 | 1.23 | 1.70 | 1.37 | 1.30 |
| **10478698** | NM_010165 | Eya2 | 3.29E-04 | 1.10 | 1.03 | 1.36 | 3.32 |
| **10366043** | NM_026268 | Dusp6 | 8.28E-06 | 1.20 | 1.11 | 1.24 | -2.45 |
| **10388718** | NM_008952 | Pipox | 4.37E-03 | 2.18 | 1.85 | 1.24 | -1.36 |
| **10505270** | NM_025286 | Slc31a2 | 8.96E-05 | -1.04 | 1.15 | 1.24 | 2.59 |
| **10440333** | NM_173069 | Speer2 | 6.39E-03 | -1.04 | 1.10 | 1.22 | 2.93 |
| **10584827** | NM_007962 | Mpzl2 | 1.67E-03 | 1.17 | 1.26 | 1.17 | 3.63 |
| **10574230** | XR_033927 | LOC676736 | 2.32E-04 | 1.19 | -1.01 | 1.15 | 2.26 |
| **10358599** | NM_001024720 | Hmcn1 | 9.76E-03 | -1.20 | 1.50 | 1.10 | 1.40 |
| **10507840** | NM_013905 | Heyl | 1.06E-04 | 1.30 | 1.98 | 1.06 | 3.54 |
| **10531261** | NM_028478 | Rassf6 | 6.08E-03 | 1.06 | -1.20 | 1.02 | 1.42 |
|  | **Gene Average** |  |  | **1.70** | **3.28** | **3.36** | **3.47** |
