# Supplementary material for: Pre-B Cell Receptor Signaling Induces Immunoglobulin κ Locus Accessibility by Functional Redistribution of Enhancer-Mediated Chromatin Interactions
Source: PLoS Biol. 2014 Feb 18;12(2):e1001791. doi: 10.1371/journal.pbio.1001791 (PMC3928034; doi:10.1371/journal.pbio.1001791)
Supplement: Table S2 — Genes up-regulated in the absence of Btk and Slp65. (DOC) [file pbio.1001791.s011.doc]

**Supplementary Table S2. Genes down regulated in the absence of Btk and Slp65.**

ANOVA analysis of genes differentially expressed between *Rag1^-/-^*pro-B cells and V_H_81X Tg *Rag1^-/-^*pre-B cells that were either wild-type (WT), *Btk^-/-^, Slp65^-/-^* or *Btk^-/-^Slp65^-/-^.*

| **ID**  **Probe set** | **Accession number** | **Gene** | **Adjusted**  **p-value** | **Fold Change**  **(Btk KO)** | **Fold Change**  **(Slp65 KO)** | **Fold Change (Btk/Slp65 KO)** | **Fold Change**  **(Rag1 KO)^a^** |
| --- | --- | --- | --- | --- | --- | --- | --- |
| 10390640 | NM_011771 | Ikzf3 | 7.05E-08 | -1.66 | -3.99 | -29.34 | -26.09 |
| 10360018 | NM_001160215 | Fcrla | 3.09E-07 | -2.15 | -3.28 | -22.50 | -15.94 |
| 10500677 | NM_013486 | Cd2 | 2.57E-04 | -4.82 | -5.35 | -20.52 | -24.26 |
| 10469278 | NM_008367 | Il2ra | 1.63E-03 | -5.21 | -8.79 | -15.90 | -16.26 |
| 10446334 | NM_133236 | Glcci1 | 3.13E-04 | -1.69 | -4.35 | -14.87 | -17.34 |
| 10375402 | NM_009616 | Adam19 | 7.03E-05 | -4.89 | -8.47 | -13.81 | -12.98 |
| 10450154 | NM_010378 | H2-Aa | 1.45E-04 | -2.04 | -5.75 | -13.16 | -19.80 |
| 10486061 | NM_011246 | Rasgrp1 | 9.01E-08 | -7.99 | -14.21 | -13.06 | -14.30 |
| 10586781 | NM_181072 | Myo1e | 1.87E-04 | -2.62 | -5.73 | -12.55 | -10.96 |
| 10489107 | NM_018851 | Samhd1 | 4.95E-06 | -2.73 | -3.43 | -11.85 | -8.93 |
| 10384020 | NM_017401 | Polm | 2.35E-06 | -1.91 | -4.17 | -10.09 | -12.25 |
| 10521678 | NM_007646 | Cd38 | 4.15E-05 | -2.53 | -2.94 | -9.41 | -7.87 |
| 10456005 | NM_001042605 | Cd74 | 4.35E-04 | -2.15 | -3.31 | -9.18 | -14.90 |
| 10441115 | NM_145125 | Brwd1 | 1.13E-04 | -4.72 | -8.99 | -8.98 | -8.02 |
| 10379630 | NM_011408 | Slfn2 | 1.11E-04 | -5.82 | -3.83 | -8.66 | -3.66 |
| 10449741 | NM_010831 | Sik1 | 4.65E-05 | -1.64 | -5.55 | -8.55 | -8.60 |
| 10483679 | NM_001080707 | Gpr155 | 2.62E-04 | -1.68 | -3.09 | -8.27 | -5.81 |
| 10406598 | NM_172588 | Serinc5 | 5.69E-04 | -2.49 | -3.64 | -7.77 | -4.29 |
| 10357488 | NM_010016 | Cd55 | 4.22E-04 | -5.52 | -3.52 | -7.14 | -4.87 |
| 10563441 | NM_010129 | Emp3 | 5.61E-03 | -2.31 | -2.84 | -6.79 | -6.48 |
| 10366546 | NM_027468 | Cpm | 1.37E-04 | -1.38 | -2.57 | -6.76 | -6.38 |
| 10420758 | NM_007549 | Blk | 7.33E-05 | -2.10 | -2.78 | -6.62 | -4.22 |
| 10404904 | NM_001081425 | Rbm24 | 1.45E-04 | -3.57 | -6.21 | -6.55 | -4.50 |
| 10404132 | NM_001111110 | Cmah | 9.25E-03 | -1.89 | -2.87 | -6.39 | -8.60 |
| 10381049 | NM_001080925 | Rapgefl1 | 1.13E-03 | -1.43 | -2.29 | -6.15 | -7.92 |
| 10381798 | NM_010858 | Myl4 | 1.07E-06 | -1.29 | -3.76 | -5.85 | -9.53 |
| 10359201 | NM_001159965 | Ralgps2 | 1.11E-06 | -1.59 | -3.40 | -5.75 | -6.21 |
| 10502510 | NM_010723 | Lmo4 | 1.73E-03 | -1.90 | -3.56 | -5.70 | -7.22 |
| 10547177 | NM_178045 | Rassf4 | 1.73E-04 | -4.34 | -4.75 | -5.65 | -3.99 |
| 10590031 | NM_133721 | Itga9 | 5.79E-05 | -2.11 | -4.37 | -5.04 | -4.99 |
| 10352000 | NM_133809 | Kmo | 7.40E-04 | -3.06 | -3.72 | -4.91 | -5.16 |
| 10350646 | NM_001039644 | Edem3 | 1.14E-04 | -3.56 | -3.86 | -4.85 | -3.92 |
| 10371379 | NM_001004363 | Nuak1 | 5.84E-04 | -2.34 | -3.14 | -4.80 | -3.79 |
| 10404389 | NM_013674 | Irf4 | 7.87E-05 | -1.60 | -2.16 | -4.75 | -5.29 |
| 10377215 | NM_008088 | Gas7 | 8.24E-04 | -1.77 | -1.80 | -4.73 | -2.79 |
| 10594840 | NM_001033208 | Gcom1 | 4.68E-05 | -3.90 | -4.04 | -4.73 | -3.81 |
| 10414113 | NM_001004436 | Wapal | 8.41E-04 | -1.00 | -2.96 | -4.61 | -4.00 |
| 10360957 | NM_172650 | Kctd3 | 5.22E-04 | -2.68 | -2.84 | -4.59 | -4.64 |
| 10564539 | NM_001024703 | Mctp2 | 3.85E-03 | -2.26 | -2.93 | -4.58 | -5.03 |
| 10438415 | ENSMUST00000103752 | Igl-V2 | 6.90E-05 | -3.41 | -3.87 | -4.57 | -4.81 |
| 10437698 | NM_029582 | Txndc11 | 1.49E-03 | -1.95 | -2.60 | -4.42 | -3.92 |
| 10381154 | NM_009923 | Cnp | 6.67E-03 | -2.00 | -2.27 | -4.36 | -4.26 |
| 10399470 | NM_144551 | Trib2 | 1.10E-05 | 1.93 | -2.37 | -4.36 | -7.67 |
| 10351224 | NM_007976 | F5 | 7.51E-05 | -2.96 | -3.03 | -4.19 | -3.41 |
| 10457787 | NM_001081403 | Klhl14 | 2.79E-03 | -2.85 | -2.17 | -4.17 | -4.49 |
| 10463945 | NM_001085390 | Dusp5 | 1.21E-03 | -3.66 | -4.83 | -4.16 | -3.34 |
| 10408280 | NM_026825 | Lrrc16a | 4.89E-04 | -3.22 | -2.99 | -4.09 | -4.97 |
| 10365845 | NM_053072 | Fgd6 | 3.70E-03 | -2.69 | -2.37 | -4.02 | -4.94 |
| 10391577 | NM_001077696 | Hdac5 | 2.64E-03 | -1.55 | -2.00 | -4.01 | -2.87 |
| 10554375 | NM_013659 | Sema4b | 1.23E-06 | -1.53 | -2.33 | -4.00 | -6.25 |
| 10438405 | M94350 | Igl-V1 | 1.58E-06 | -3.42 | -3.07 | -3.98 | -6.20 |
| 10486112 | NM_138313 | Bmf | 7.87E-03 | -1.21 | -1.77 | -3.97 | -4.73 |
| 10522182 | NM_001081105 | Rhoh | 1.83E-03 | -1.87 | -2.82 | -3.95 | -3.67 |
| 10447341 | NM_145491 | Rhoq | 2.60E-03 | -1.11 | -1.34 | -3.94 | -4.82 |
| 10444298 | NM_010382 | H2-Eb1 | 2.53E-04 | -1.71 | -3.86 | -3.93 | -3.92 |
| 10606694 | NM_013482 | Btk | 2.81E-06 | -3.14 | 1.01 | -3.92 | -1.02 |
| 10361139 | NM_011633 | Traf5 | 8.72E-06 | -2.15 | -2.64 | -3.87 | -4.53 |
| 10349593 | NM_026976 | Faim3 | 6.26E-03 | -2.91 | -3.04 | -3.86 | -3.47 |
| 10416423 | NM_198642 | 5031414D18Rik | 1.64E-06 | -1.54 | -2.04 | -3.64 | -3.79 |
| 10567702 | NM_144529 | Arhgap17 | 1.64E-05 | -1.49 | -2.54 | -3.61 | -5.02 |
| 10477250 | NM_010407 | Hck | 2.41E-04 | -3.24 | -3.22 | -3.58 | -3.38 |
| 10572466 | NM_201607 | Pde4c | 1.74E-03 | -2.22 | -4.28 | -3.48 | -3.24 |
| 10407126 | NM_152804 | Plk2 | 3.34E-03 | 1.58 | -3.27 | -3.42 | -6.28 |
| 10400405 | NM_010907 | Nfkbia | 1.40E-03 | -1.44 | -2.61 | -3.40 | -5.78 |
| 10439092 | BC060601 | 1700021K19Rik | 1.82E-04 | -2.29 | -2.60 | -3.39 | -3.72 |
| 10439239 | NM_153550 | Dirc2 | 1.33E-04 | -2.12 | -3.02 | -3.36 | -4.35 |
| 10494595 | NM_010928 | Notch2 | 3.09E-03 | -3.28 | -3.25 | -3.36 | -3.94 |
| 10404941 | NM_172262 | Aof1 | 2.47E-04 | -2.05 | -2.10 | -3.35 | -3.31 |
| 10503695 | NM_007521 | Bach2 | 1.02E-04 | -1.19 | -1.97 | -3.34 | -5.27 |
| 10569707 | NM_001093765 | Myadm | 4.47E-03 | -2.53 | -1.78 | -3.31 | -2.98 |
| 10389617 | NM_177167 | Ppm1e | 9.38E-04 | -1.59 | -1.59 | -3.20 | -2.47 |
| 10523579 | NM_029270 | Arhgap24 | 9.54E-03 | -1.77 | -1.92 | -3.17 | -3.40 |
| 10562812 | NM_019866 | Spib | 3.88E-04 | -1.57 | -1.94 | -3.16 | -3.22 |
| 10417027 | NM_021386 | Cldn10 | 3.03E-03 | -1.95 | -2.35 | -3.11 | -2.70 |
| 10381588 | NM_008175 | Grn | 4.21E-05 | -1.25 | -1.53 | -3.05 | -3.47 |
| 10364559 | NM_007880 | Arid3a | 1.13E-03 | -1.80 | -2.16 | -3.04 | -3.18 |
| 10378649 | NM_173388 | Slc43a2 | 1.23E-03 | -1.69 | -2.07 | -2.95 | -3.32 |
| 10594001 | NM_019689 | Arid3b | 5.74E-04 | -1.79 | -2.51 | -2.91 | -3.53 |
| 10405211 | NM_011817 | Gadd45g | 5.11E-04 | -2.21 | -2.56 | -2.89 | -2.74 |
| 10354031 | NM_207228 | Tsga10 | 4.32E-03 | -1.69 | -2.37 | -2.88 | -2.44 |
| 10444665 | NM_016765 | Ddah2 | 5.88E-03 | -1.09 | -1.73 | -2.86 | -2.67 |
| 10405216 | NM_011518 | Syk | 1.06E-03 | -1.60 | -2.29 | -2.82 | -3.34 |
| 10586844 | NM_007399 | Adam10 | 1.58E-03 | -1.51 | -2.01 | -2.82 | -2.51 |
| 10388211 | NM_029932 | Spns3 | 3.01E-04 | -1.37 | -2.03 | -2.78 | -5.17 |
| 10435443 | NM_175111 | Hspbap1 | 2.03E-03 | -1.74 | -2.06 | -2.77 | -3.18 |
| 10509122 | NM_009924 | Cnr2 | 3.24E-05 | -3.68 | -2.12 | -2.75 | -3.92 |
| 10512470 | NM_001110320 | Cd72 | 2.17E-03 | 1.04 | -1.52 | -2.73 | -2.76 |
| 10561008 | NM_001039185 | Ceacam1 | 4.31E-03 | -1.50 | -2.01 | -2.70 | -4.49 |
| 10591281 | NM_016919 | Col5a3 | 5.66E-04 | -2.25 | -2.65 | -2.68 | -2.11 |
| 10467887 | NM_030703 | Cpn1 | 8.02E-03 | -2.43 | -2.40 | -2.68 | -2.76 |
| 10381187 | NM_016920 | Atp6v0a1 | 1.07E-04 | -1.66 | -2.17 | -2.67 | -4.03 |
| 10479379 | NM_148933 | Slco4a1 | 5.55E-03 | -1.64 | -2.14 | -2.66 | -2.93 |
| 10409502 | NM_013739 | Dok3 | 2.32E-03 | -1.26 | -2.02 | -2.63 | -3.73 |
| 10396936 | NM_001146217 | Smoc1 | 2.18E-04 | -2.17 | -3.00 | -2.62 | -2.21 |
| 10568553 | NM_029935 | Chst15 | 1.73E-03 | -1.43 | -1.92 | -2.62 | -3.32 |
| 10509838 | NM_008812 | Padi2 | 6.23E-03 | -1.84 | -2.47 | -2.59 | -2.89 |
| 10405179 | NM_010101 | S1pr3 | 6.77E-03 | -1.87 | -2.85 | -2.56 | -2.39 |
| 10439346 | NM_001113401 | Eaf2 | 3.95E-03 | -1.75 | -2.02 | -2.56 | -1.90 |
| 10503180 | NM_001081417 | Chd7 | 6.02E-03 | -2.07 | -1.17 | -2.55 | -1.71 |
| 10488655 | NM_009743 | Bcl2l1 | 2.30E-04 | -1.31 | -2.01 | -2.53 | -2.97 |
| 10557399 | NM_145587 | Sbk1 | 2.07E-03 | 1.02 | -1.47 | -2.53 | -3.24 |
| 10422312 | NM_023878 | Cldn10 | 2.24E-03 | -1.69 | -1.90 | -2.52 | -2.18 |
| 10359982 | NM_001081304 | Atf6 | 3.05E-04 | -1.46 | -1.94 | -2.50 | -3.07 |
| 10350977 | NM_001162896 | 4930523C07Rik | 1.67E-03 | -1.56 | -1.76 | -2.49 | -2.69 |
| 10543118 | NM_133236 | Glcci1 | 8.62E-04 | -1.67 | -2.21 | -2.49 | -2.09 |
| 10503098 | NM_001111096 | Lyn | 1.35E-04 | -1.53 | -1.54 | -2.47 | -2.67 |
| 10354085 | NM_019570 | Rev1 | 2.52E-04 | -1.53 | -1.66 | -2.46 | -2.67 |
| 10350733 | NM_011267 | Rgs16 | 7.74E-03 | -2.24 | -2.70 | -2.44 | -2.79 |
| 10592535 | NM_011436 | Sorl1 | 8.41E-03 | -1.43 | -2.34 | -2.43 | -2.81 |
| 10606102 | NM_008832 | Phka1 | 1.88E-03 | -1.28 | -1.68 | -2.40 | -3.26 |
| 10404774 | NM_007772 | Hivep1 | 7.71E-06 | -2.18 | -1.85 | -2.37 | -2.41 |
| 10431962 | NM_008902 | Pp11r | 4.49E-03 | -1.09 | -1.23 | -2.35 | -2.24 |
| 10529801 | NM_001159963 | Fbxl5 | 6.25E-04 | -1.45 | -2.03 | -2.34 | -2.84 |
| 10570236 | NM_178076 | Mcf2l | 6.56E-03 | -1.39 | -1.72 | -2.26 | -1.81 |
| 10552380 | NM_172900 | Siglecg | 1.59E-03 | -1.24 | -1.67 | -2.25 | -3.54 |
| 10549506 | NM_177192 | Dennd5b | 3.23E-03 | -1.01 | -1.56 | -2.23 | -3.58 |
| 10581996 | NM_029441 | Cdyl2 | 8.79E-04 | -1.35 | -1.96 | -2.22 | -3.08 |
| 10512640 | NM_015828 | Gne | 2.63E-03 | -2.19 | -1.98 | -2.22 | -2.54 |
| 10405163 | NM_146043 | Spin1 | 2.99E-04 | -1.77 | -1.94 | -2.20 | -2.12 |
| 10401160 | BC066067 | 6330442E10Rik | 2.85E-04 | -1.15 | -1.22 | -2.19 | -2.20 |
| 10426093 | NM_181412 | Zbed4 | 5.50E-03 | -1.43 | -1.80 | -2.18 | -2.48 |
| 10449672 | NM_080727 | Tmprss3 | 1.96E-03 | 1.58 | -1.32 | -2.18 | -3.34 |
| 10600284 | NM_019684 | Srpk3 | 3.37E-03 | -1.03 | -1.33 | -2.18 | -2.48 |
| 10398907 | NM_178911 | Pld4 | 2.23E-05 | -1.24 | -1.03 | -2.17 | -5.60 |
| 10363762 | NM_177794 | Tmem26 | 3.94E-03 | -2.09 | -1.89 | -2.17 | -2.32 |
| 10606088 | NM_027382 | Hdac8 | 1.55E-03 | -1.48 | -1.76 | -2.16 | -2.13 |
| 10409684 | ENSMUST00000022032 | 2210016F16Rik | 8.22E-03 | -1.48 | -1.46 | -2.16 | -2.74 |
| 10588479 | NM_031178 | Tlr9 | 8.81E-05 | -1.57 | -1.81 | -2.15 | -3.38 |
| 10557326 | NM_001008700 | Il4ra | 2.37E-03 | -1.58 | -1.88 | -2.15 | -2.23 |
| 10467068 | NM_144792 | Sgms1 | 3.22E-04 | -1.17 | -1.61 | -2.15 | -2.59 |
| 10454229 | NM_001081403 | Klhl14 | 4.51E-04 | -2.00 | -2.03 | -2.15 | -2.46 |
| 10409076 | NM_011078 | Phf2 | 1.49E-03 | -1.60 | -1.78 | -2.15 | -3.17 |
| 10484894 | NM_008982 | Ptprj | 9.08E-05 | -1.05 | -1.15 | -2.14 | -2.87 |
| 10554370 | NM_175433 | Zfp710 | 9.03E-03 | -1.50 | -1.64 | -2.14 | -2.99 |
| 10437687 | NM_019980 | Litaf | 2.13E-03 | -1.61 | -1.39 | -2.09 | -1.10 |
| 10431974 | NM_144850 | Rapgef3 | 1.15E-03 | 1.12 | -1.77 | -2.09 | -2.46 |
| 10365971 | NM_007569 | Btg1 | 3.39E-04 | -1.06 | -1.74 | -2.06 | -2.58 |
| 10447294 | NM_011104 | Prkce | 4.39E-03 | -1.53 | -2.18 | -2.05 | -3.08 |
| 10392183 | NM_023913 | Ern1 | 9.13E-03 | -1.65 | -1.75 | -2.05 | -1.74 |
| 10363445 | NM_028732 | 4632428N05Rik | 8.20E-04 | -2.18 | -2.02 | -2.02 | -1.36 |
| 10374333 | NM_001025597 | Ikzf1 | 5.19E-05 | -1.31 | -1.77 | -1.99 | -2.44 |
| 10562132 | NM_001043317 | Cd22 | 3.32E-05 | 1.29 | 1.14 | -1.98 | -6.06 |
| 10434291 | NM_001159407 | B3gnt5 | 9.15E-03 | -1.10 | -1.08 | -1.98 | -2.98 |
| 10596200 | NM_178638 | Tmem108 | 1.10E-04 | 1.57 | -1.31 | -1.97 | -3.56 |
| 10508663 | NM_010686 | Laptm5 | 9.84E-03 | -1.32 | -1.18 | -1.97 | -1.89 |
| 10437443 | BC004063 | 5730403B10Rik | 3.75E-03 | -1.55 | -1.62 | -1.96 | -1.90 |
| 10461765 | NM_134152 | Lpxn | 6.26E-03 | -1.36 | -1.16 | -1.93 | -3.52 |
| 10512129 | NM_022305 | B4galt1 | 1.59E-04 | -1.31 | -1.33 | -1.90 | -2.10 |
| 10560202 | NM_001081418 | Gltscr1 | 1.85E-03 | -1.29 | -1.38 | -1.87 | -3.43 |
| 10572679 | NM_146211 | Glt25d1 | 6.86E-03 | -1.04 | -1.64 | -1.86 | -2.25 |
| 10560964 | NM_011138 | Pou2f2 | 6.29E-03 | -1.24 | -1.30 | -1.84 | -2.56 |
| 10349834 | BC132141 | Pik3c2b | 1.20E-03 | -1.65 | -1.82 | -1.84 | -2.87 |
| 10565018 | NM_016721 | Iqgap1 | 1.84E-03 | -1.35 | -1.71 | -1.81 | -2.09 |
| 10501286 | NM_175183 | Atxn7l2 | 7.93E-03 | -1.44 | -1.46 | -1.80 | -2.81 |
| 10462140 | NM_028785 | Dock8 | 7.39E-03 | -1.89 | -1.76 | -1.80 | -2.16 |
| 10562130 | NM_194057 | Ffar1 | 4.36E-03 | -1.10 | -1.36 | -1.80 | -1.97 |
| 10569733 | NM_133962 | Arhgef18 | 3.30E-03 | -1.42 | -1.64 | -1.80 | -3.59 |
| 10495270 | NM_146137 | Amigo1 | 3.97E-03 | -1.58 | -1.77 | -1.76 | -1.58 |
| 10476237 | NM_028306 | Hspa12b | 5.61E-03 | -1.68 | -1.49 | -1.75 | 1.14 |
| 10518132 | NM_001081355 | Prdm2 | 7.08E-04 | -1.57 | -1.42 | -1.74 | -1.79 |
| 10467979 | NM_009127 | Scd1 | 1.28E-03 | 1.12 | 1.31 | -1.73 | -5.33 |
| 10435094 | NM_016788 | Tnk2 | 1.06E-04 | -1.27 | -1.20 | -1.69 | -2.95 |
| 10594785 | NM_033604 | Rnf111 | 5.91E-03 | -1.50 | -1.56 | -1.67 | -2.46 |
| 10593966 | NM_007783 | Csk | 1.36E-03 | -1.12 | -1.35 | -1.64 | -2.46 |
| 10419542 | NM_001033271 | Tmem55b | 6.53E-04 | -1.19 | -1.39 | -1.61 | -2.01 |
| 10551080 | NM_027882 | Cic | 2.64E-03 | -1.19 | -1.33 | -1.56 | -3.38 |
| 10603627 | NM_175045 | Bcor | 2.34E-03 | -1.12 | -1.17 | -1.53 | -2.15 |
| 10453918 | NM_029623 | 3110002H16Rik | 5.34E-03 | -1.23 | -1.35 | -1.52 | -1.99 |
| 10493711 | NM_028881 | Crtc2 | 9.16E-04 | -1.24 | -1.26 | -1.47 | -2.60 |
| 10464932 | NM_008451 | Klc2 | 8.23E-03 | -1.17 | -1.21 | -1.42 | -1.84 |
| 10540622 | NM_030178 | Brpf1 | 9.10E-03 | -1.23 | -1.38 | -1.40 | -1.95 |
| 10478778 | NM_001085495 | Arfgef2 | 2.75E-03 | -1.24 | -1.45 | -1.40 | -1.96 |
| 10424584 | NM_001081066 | Dennd3 | 6.36E-05 | -1.59 | -1.45 | -1.39 | -3.25 |
| 10402994 | --- |  | 3.26E-03 | -1.22 | -1.03 | -1.38 | -9.24 |
| 10572212 | NM_198101 | Gmip | 1.02E-03 | -1.22 | -1.19 | -1.38 | -2.97 |
| 10493758 | NM_139304 | Gatad2b | 9.25E-03 | -1.20 | -1.26 | -1.35 | -1.85 |
| 10516507 | NM_177758 | Zscan20 | 6.61E-05 | 1.13 | -1.10 | -1.34 | -1.49 |
| 10461475 | NM_172302 | Cpsf7 | 1.38E-03 | -1.17 | -1.24 | -1.32 | -2.05 |
| 10446172 | NM_144858 | Dus3l | 1.44E-03 | -1.10 | -1.20 | -1.27 | -1.88 |
| 10460706 | NM_028769 | Syvn1 | 8.29E-03 | 1.03 | -1.14 | -1.25 | -2.18 |
| 10465649 | NM_007928 | Mark2 | 5.82E-05 | -1.14 | -1.19 | -1.23 | -2.51 |
| 10517090 | NM_001080819 | Arid1a | 3.69E-03 | -1.05 | -1.25 | -1.21 | -2.16 |
| 10433185 | NM_009267 | Spt1 | 1.72E-03 | -1.10 | -1.08 | -1.16 | 1.52 |
| 10493177 | NM_133665 | Mef2d | 1.88E-04 | -1.11 | -1.29 | -1.15 | -2.34 |
| 10387699 | NM_153788 | Centb1 | 1.04E-03 | 1.29 | -1.09 | -1.13 | -2.86 |
| 10468691 | NM_178688 | Ablim1 | 4.66E-05 | -1.27 | -1.11 | -1.13 | 2.24 |
| 10551185 | NM_011577 | Tgfb1 | 4.48E-03 | 1.04 | -1.03 | -1.12 | -1.60 |
| 10580957 | NM_172758 | Slc38a7 | 7.81E-04 | -1.05 | 1.17 | -1.12 | -1.37 |
| 10429160 | NM_009177 | St3gal1 | 3.34E-03 | -1.01 | -1.08 | -1.11 | -2.03 |
| 10556616 | NM_001105252 | Tmc5 | 3.76E-03 | 3.00 | 1.76 | -1.06 | -1.46 |
| 10580516 | XR_031896 | Gm6625 | 1.55E-03 | 1.08 | -1.02 | -1.06 | 2.03 |
| 10483698 | NM_153138 | Wipf1 | 8.88E-03 | 1.12 | 1.37 | -1.00 | -1.55 |
|  | **Gene Average** |  |  | **-1.65** | **-2.29** | **-3.79** | **-4.15** |
