# Supplementary material for: Pre-B Cell Receptor Signaling Induces Immunoglobulin κ Locus Accessibility by Functional Redistribution of Enhancer-Mediated Chromatin Interactions
Source: PLoS Biol. 2014 Feb 18;12(2):e1001791. doi: 10.1371/journal.pbio.1001791 (PMC3928034; doi:10.1371/journal.pbio.1001791)
Supplement: Table S3 — Binding of E2a and Ikaros to the κ enhancers and Vκ genes in wild-type and Btk or Slp65-deficient pre-B cells. (DOC) [file pbio.1001791.s012.doc]

**Supplementary Table S3. Chromatin immunoprecipitation (ChIP) results** Enrichments for E2A and Ikaros as measured by quantitative PCR after ChIP on V_H_81X Tg *Rag1^-/-^*pre-B cells that were either wild-type (WT), *Btk^-/-^, Slp65^-/-^* or *Btk^-/-^Slp65^-/-^.*

| **Antibody** | **Genomic location** | | **Fold enrichment***  **(WT)** | **Fold enrichment***  **(Btk^-/-^)** | **Fold enrichment***  **(Slp65^-/-^)** | **Fold enrichment***  **(Btk^-/-^ Slp65^-/-^)** |
| --- | --- | --- | --- | --- | --- | --- |
| **E2A polyclonal** | | 3’Eκ | 43.5 | 35.3 | 26.1 | 35.1 |
|  | | iEκ | 26.0 | 10.0 | 13.8 | 6.5 |
|  | | V_κ_ 9-120 | 4.6 | 1.9 | 1.9 | 1.4 |
|  | | V_κ_ 10-96 | 17.8 | 3.8 | 4.8 | 0.7 |
|  | | V_κ_ 8-24 | 9.3 | 3.1 | 1.3 | 1.1 |
|  | |  |  |  |  |  |
| **Ikaros polyclonal** | | 3’Eκ | 6.2 | 8.2 | 1.7 | n.d. |
|  | | iEκ | 2.4 | 3.1 | 1.2 | 1.5 |
|  | | V_κ_ 9-120 | 1.8 | 1.8 | 1.1 | 1.2 |
|  | | V_κ_ 10-96 | 3.0 | 1.7 | 0.9 | 1.0 |
|  | | V_κ_ 8-24 | 1.8 | 1.9 | 0.5 | 1.0 |

*****Enrichments were calculated over a negative control region (amylase promoter region). Back-

ground levels were assessed using a non-specific control antibody (species-matched IgG) and

ranged from 0.8-1.2 fold. n.d.: not determined
